# Multi-omics uncovers transcriptional programs of gut-resident memory CD4^+^ T cells in Crohn’s disease

**DOI:** 10.1084/jem.20242106

**Published:** 2025-09-04

**Authors:** Mitsuru Arase, Mari Murakami, Takako Kihara, Ryuichi Kuwahara, Hironobu Toyota, Naoki Sumitani, Naohiko Kinoshita, Kelvin Y. Chen, Takehito Yokoi, Daisuke Motooka, Daisuke Okuzaki, Yuhe Zhao, Hazuki Miyazaki, Takayuki Ogino, Seiichi Hirota, Hiroki Ikeuchi, Kiyoshi Takeda

**Affiliations:** 1 Department of Microbiology and Immunology, Graduate School of Medicine, The University of Osaka, Osaka, Japan; 2 Immunology Frontier Research Center, The University of Osaka, Osaka, Japan; 3 Department of Diagnostic Pathology, Hyogo Medical University School of Medicine, Nishinomiya, Japan; 4 Division of Inflammatory Bowel Disease Surgery, Department of Gastroenterological Surgery, Hyogo Medical University, Nishinomiya, Japan; 5 Department of Experimental Immunology, Immunology Frontier Research Center, The University of Osaka, Osaka, Japan; 6 Department of Pediatrics, Graduate School of Medicine, The University of Osaka, Osaka, Japan; 7 Center for Infectious Disease Education and Research, The University of Osaka, Osaka, Japan; 8 Genome Information Research Center, Research Institute for Microbial Diseases, The University of Osaka, Osaka, Japan; 9 Integrated Frontier Research for Medical Science Division, Institute for Open and Transdisciplinary Research Initiatives, The University of Osaka, Osaka, Japan; 10 Department of Gastroenterological Surgery, Graduate School of Medicine, The University of Osaka, Osaka, Japan

## Abstract

Tissue-resident memory T cells (T_RM_) remain in nonlymphatic barrier tissues for extended periods and are deeply involved in immune memory at the site of inflammation. Here, we employed multilayered single-cell analytic approaches including chromatin, gene, and protein profiling to characterize a unique CD4^+^ T_RM_ subset present in the inflamed gut mucosa of Crohn’s disease patients. We identified two key transcription factors, RUNX2 and BHLHE40, as regulators of pathologically relevant CD4^+^ T_RM_. These transcriptional regulators work together to induce distinct cellular properties of disease-specific T_RM_, such as cytotoxicity, T helper 1–effector activity, and tissue retention. Downregulation of RUNX2 and BHLHE40 in patient-derived gut CD4^+^ T cells resulted in the mitigation of the pathogenic phenotype of these cells. Conversely, the ectopic overexpression of both transcription factors in healthy donor–derived CD4^+^ T cells drove IFN-γ pathways and enhanced tissue residency. Our findings illuminate the transcriptional programs driving disease-specific T cell formation in Crohn’s disease.

## Teaser

Multiple omics analyses identified RUNX2 and BHLHE40 as key factors driving T_RM_ characteristic of Crohn’s disease. The overexpression of these transcription factors induced Crohn’s disease–specific T_RM_, and blocking these transcription factors mitigated the cytotoxic T_RM_ phenotype in human disease samples.

## Introduction

The cellular properties of tissue-resident memory T cells (T_RM_) are a double-edged sword; their long-term residence in peripheral tissues, coupled with their immune memory and effector activity, allows them to serve as the front line of host defense, while their dysregulated immune responses can lead to tissue inflammation and contribute to the development of pathogenic conditions (Murakami, 2024). For medical intervention in chronic and relapsing gastrointestinal inflammation that characterizes inflammatory bowel disease (IBD), targeting T_RM_, which are responsible for long-term local immune recall, might be a promising approach. The differentiation of T_RM_ involves various cues from the tissue microenvironment, contributing to their specialized functions and localization within specific tissues, and leading to a high diversity of T_RM._ Assessing the role of the human T_RM_ in disease *in situ* is challenging, particularly in visceral organs such as the intestine, thus inferred by correlative studies. Previous reports, including ours, have demonstrated that specific T_RM_ subsets are associated with various conditions of IBD by inducing inflammation, or, conversely, exerting tissue-protective effects in some cases (Lamb et al., 2017; Bishu et al., 2019; Roosenboom et al., 2019; Jaeger et al., 2021; Yokoi et al., 2023).

Advances in high-throughput sequencing technology enable us to visualize highly diverse immune cell populations that would otherwise not be apparent. This allows for the identification of immune cell subsets specifically induced in the disease context. We previously reported that a subset of CD4^+^ T_RM_ appears in the affected lesion of Crohn’s disease (CD) patients (Yokoi et al., 2023). These cells are poised for rapid activation under the gut microenvironment of patients with CD, even without exogenous T cell receptor (TCR) ligation, secreting high levels of T helper cell 1 (Th1)–type cytokines and cytotoxic molecules. This T cell subset expresses high levels of CD103, which is the αE subunit of αEβ7 integrin, a cell surface marker of T_RM_. A high frequency of disease-predominant CD4^+^ T_RM_ in the gut mucosa is inversely correlated with favorable prognosis in patients with CD, suggesting that the accumulation of these cells is a major hallmark of CD pathogenesis. Understanding the mechanisms underlying the induction and maintenance of these pathologically relevant immune memory cells might be one of the most important issues for developing targeted therapeutic strategies for CD, yet many questions remain unaddressed. Recent studies have elucidated the transcriptional mechanisms underlying the differentiation, maintenance, and function of T_RM_, particularly CD8^+^ T_RM_. Hobit and Blimp1 govern the transcriptional program of the CD8^+^ T_RM_ by repressing the genes associated with tissue egress, such as *Klf2*, *S1pr1*, and *Ccr7* (Mackay et al., 2016). In addition, the expression and activity of the T-box transcription factor (TF) Eomesodermin (Eomes) and its related homolog T-bet are tightly regulated during T_RM_ development (Laidlaw et al., 2014; Mackay et al., 2015). Both T-box TFs decrease with T_RM_ maturation, and Eomes is lost in the final stage, but the sustained low levels of T-bet are required for T_RM_ responsiveness to IL-15, which induces Hobit. Runt-related TF 3 (RUNX3) is an important transcriptional regulator associated with the differentiation and maintenance of the CD8^+^ T_RM_ (Milner et al., 2017). However, the expression of RUNX3 is repressed in CD4^+^ T cells by the CD4^+^ lineage–specific TF T helper–inducing POZ/Krüppel-like factor (ThPOK). This renders CD4^+^ T cells unresponsive to TGF-β, which is required for CD8^+^ T_RM_ formation (Fonseca et al., 2022). These findings collectively indicate that the formation of CD4^+^ and CD8^+^ T_RM_ is tightly regulated by distinct mechanisms. The multifaceted nature of T_RM_ is therefore closely linked to local signaling cues within their diverse environments, correlating with altered chromatin accessibility and differential dependence on transcriptional regulators.

Here, we elucidated the molecular mechanisms involved in the pathogenesis of CD by focusing on the CD-specific CD4^+^ T_RM_ that we previously reported. Comprehensive analyses on gene expression, proteins, and open chromatin regions at a single-cell level corroborated and extended our previous findings. We identified RUNX2 and BHLHE40 as two key molecules in the transcriptional governance of disease-specific CD4^+^ T_RM_ present in the gut mucosa of CD patients. Elucidating the mechanism by which this CD-specific T cell subset is induced in the pathological context of CD will pave the way for decoding the molecular basis of CD.

## Results

### A dual assay of the transcriptome and proteome profiles CD-associated T_RM_

**Figure 1.**
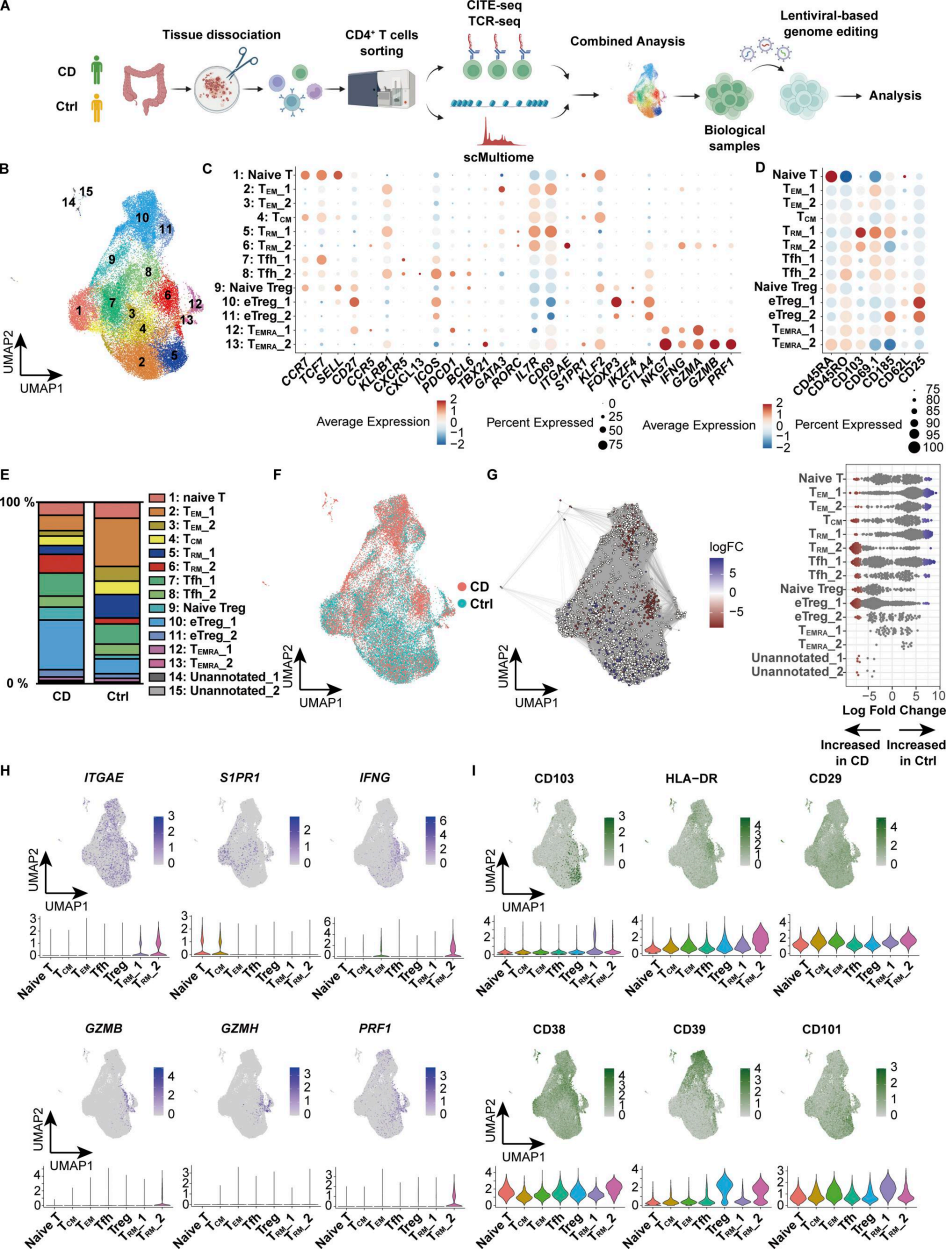
**Characterization of CD4** ^**+**^ **T cells in the colon lamina propria by CITE-seq. (A)** Schematic overview of the entire experimental workflow covering all procedures in Figs. 1–8. **(B)** UMAP plot visualizing colonic lamina propria CD4^+^ T cells from CD (*n* = 3, Patient 1–3 in Table S1) and control (*n* = 3, Ctrl-Patient 1–3 in Table S1) samples. Annotations are shown in C. **(C and D)** Expression levels of selected RNA (C) and protein (D) markers across identified clusters. The dot size indicates the percentage of cells expressing the gene within each cluster, while color intensity reflects average expression level. **(E)** Cluster proportion across disease conditions. Each bar represents 100% of cells from a given disease, with segments denoting the relative abundance of each cluster. **(F)** Distribution of CD-derived (red) and control-derived (blue) cells. **(G)** Left: Graph representation of neighborhoods (Nhoods) identified by Milo. Nodes represent Nhoods, colored by their log_2_ FC between CD and control samples. Nondifferential abundance Nhoods (FDR ≥ 0.1) are white, and sizes correspond to cell number within a Nhood. Edges depict shared cells between adjacent Nhoods. Right: Beeswarm plot displaying adjusted log_2_ FC distribution in abundance between CD and control samples within Nhoods, stratified by 15 cell types. Colors match the UMAP. (**H and I)** Feature plots showing the expression of key marker genes (H) and proteins (I) that characterize the T_RM__2 cluster. Color intensity represents expression levels, with darker shades indicating higher expression. FC, fold change.

**Figure S1.**
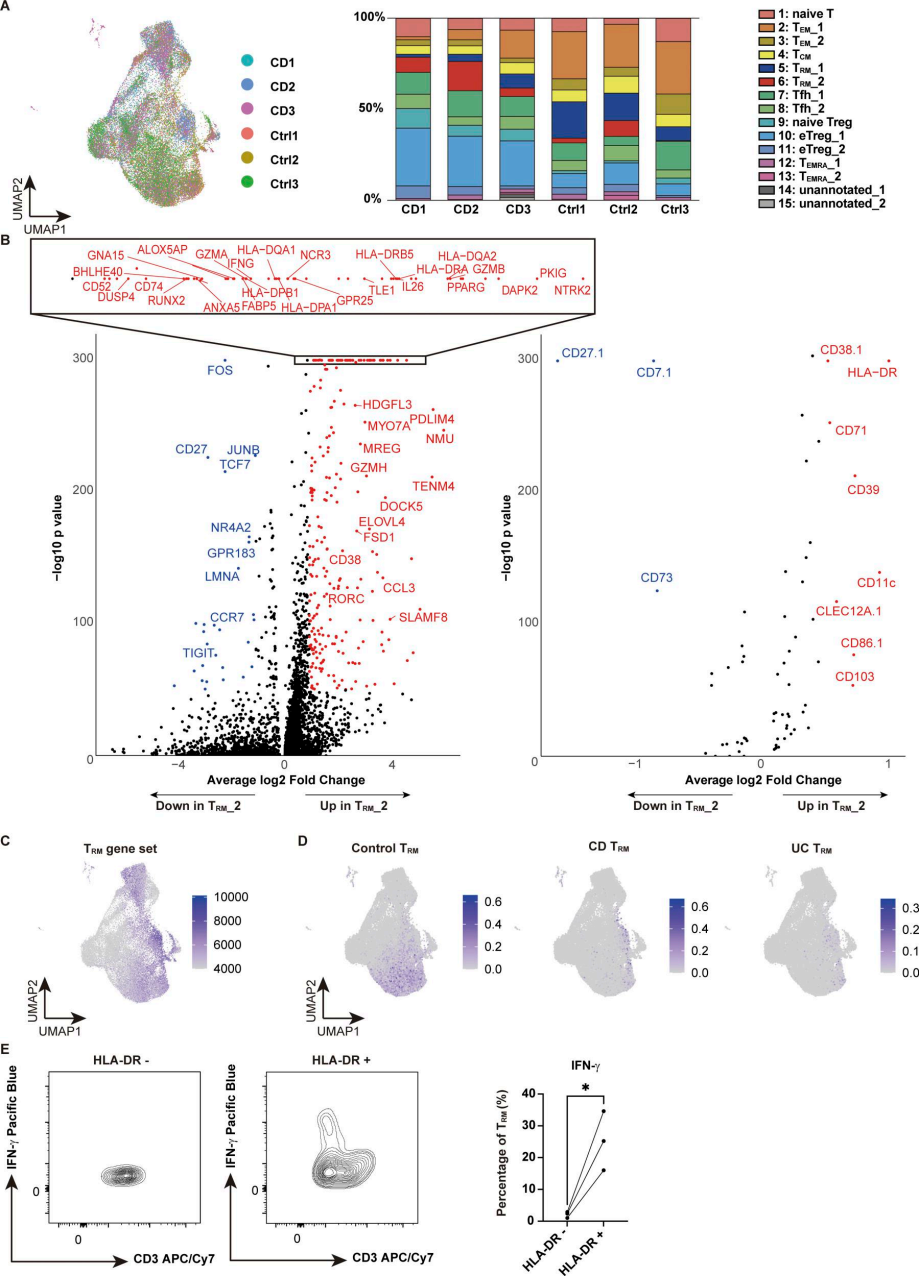
**Proportion of each sample in CITE-seq and RNA and protein expression of T**_**RM**_ **_2. (A)** UMAP (left) and bar chart (right) showing the proportion of each sample (CD: CD Patient 1–3; Control: Ctrl-Patient 1–3 in Table S1). **(B)** Volcano plot depicting differentially expressed genes (left) and proteins (right) in the T_RM__2 cluster. **(C)** Single-cell GSEA using gene sets from activated CD4^+^ T_RM_ in published dataset (GSE126030). **(D)** Reference mapping using scRNA-seq data from prior dataset (GSE218000) showing T_RM_ from control (left), CD (center), and ulcerative colitis (UC) (right). **(E)** Flow cytometry comparison of IFN-γ expression in HLA-DR–negative (HLA-DR^−^) and HLA-DR–positive (HLA-DR^+^) cells among CD3^+^ CD4^+^ CD103^+^ T cells from CD patients. *n* = 3 per group. Statistical significance for the comparisons was determined using a paired *t* test. *P < 0.05. UC, ulcerative colitis.

Single-cell profiling of immune cells provides powerful information in the interpretation of immunological disorders, but to date, most single-cell databases on diseases have been built from RNA-sequencing (RNA-seq) data, and more comprehensive datasets at the epigenetic and protein levels are required. Thus, to create transcriptional and translational atlas of the colonic mucosa in patients with CD, we performed cellular indexing of transcriptomes and epitopes by sequencing (CITE-seq) with an antibody panel comprising 130 epitopes on colonic CD4^+^ T cells isolated from the inflamed mucosa of CD patients and unaffected mucosa of colorectal cancer patients (controls) (Table S1). We profiled a total of 34,715 cells, with 18,755 and 15,960 for colorectal cancer control and CD, respectively (Fig. 1 A). Following batch effect correction of the data by the anchor-based reciprocal principal component analysis (RPCA) algorithm, we projected cells in two dimensions using Uniform Manifold Approximation and Projection (UMAP) based on the transcriptome analysis, with the cell surface protein expression overlaid. Each subset was annotated by unique transcriptional signatures revealed by differentially expressed genes and proteins between the subclusters (Fig. 1, B–D; and Table S2). Among all CD4^+^ T cell subsets, one of the CD4^+^ T_RM_ clusters, T_RM__2 (cluster 6), and effector regulatory T cells (eTreg_1: cluster 10) were predominantly expressed in CD patients, suggesting potential local interactions in the inflamed tissue, while GATA3-expressing effector memory T cells (T_EM__1: cluster 2) were decreased (Fig. 1, E and F; and Fig. S1 A). CD4^+^ T_RM_, characterized by the high expression of CD103, CD69, and *ITGAE* along with the decreased expression of the tissue-egress marker *S1PR1* (Fig. 1, C, D, H, and I; and Fig. S1 B), were clustered into two distinct subsets, T_RM__1 and T_RM__2. Milo, a computational framework for differential abundance testing without relying on cell clustering (Dann et al., 2022), overcomes limitations on adequate resolution and continuous trajectories of the clustering method. Applying Milo to our dataset revealed that most of the neighborhoods comprising T_RM__2 were highly enriched in CD, while those of T_RM__1 were predominant in controls (Fig. 1 G). To validate their T_RM_ identity, we compared our clusters with T_RM_ gene signatures derived from a previously published human single-cell RNA-seq (scRNA-seq) dataset (GSE126030) (Szabo et al., 2019). Gene set enrichment analysis (GSEA) showed that T_RM_ signatures mapped to both T_RM__1 and T_RM__2 (Fig. S1 C). In line with our previous report (Yokoi et al., 2023), the CD-predominant T_RM__2 exhibited the high expression of *IFNG*, *GZM*s, and *PRF1* (Fig. 1, C and H; and Fig. S1 B). To relate T_RM__2 to the CD-specific T_RM_ from our earlier study, we integrated the current dataset with our previously published single-cell transcriptomic dataset of colonic CD4^+^ T cells from patients with IBD and controls (GSE218000). Specifically, we projected the prior data onto UMAP generated from the current CITE-seq dataset by multimodal reference mapping, a method to integrate single-cell datasets across modalities (Hao et al., 2024) (Fig. S1 D). In this integrated analysis, CD-derived CD4^+^ T_RM_ in our prior study were primarily mapped to T_RM__2, whereas control-derived CD4^+^ T_RM_ were distributed across T_RM__1 and a portion of T_EM__2. In contrast, ulcerative colitis–derived T_RM_ were scarcely detected on the current UMAP. These findings indicate that the CD-associated T_RM_ subset reported previously corresponds to the T_RM__2 identified in the present study.

Immunophenotypic characterization of T_RM__2 may provide further insights into the molecular features of this subset. CD103, CCR5, and CD161, which we employed as surrogate markers for the isolation of T_RM_ abundant in CD, were indeed highly expressed in T_RM__2 (Yokoi et al., 2023). However, while CD103 is highly specific, CCR5 and CD161 (encoded by *KLRB1*) lack sufficient specificity (Fig. 1 C). Therefore, identification of a set of cell surface markers to reliably isolate disease-specific inflammatory T_RM_ has remained a significant challenge. Leveraging our comprehensive cell surface protein expression analyses, T_RM__2 was found to specifically express HLA-DR in CD4^+^ T cells, which was also pronounced at the transcriptional level, suggesting that it may be useful as a marker to segregate T_RM__1 and T_RM__2 (Fig. 1 I and Fig. S1 B). CD29 (integrin β1), which forms very late antigen 1 (VLA1) as a partner protein of CD49a (integrin α1), was considerably enriched in T_RM__2 compared to T_RM__1, consistent with a previous report showing that CD29 identifies human polyfunctional CD4^+^ T cells with cytotoxic gene expression (Nicolet et al., 2021). Additionally, CD38 and CD39 were highly co-expressed in a fraction of T_RM__2 but were poorly expressed in T_RM__1. Previous work has demonstrated that the high expression of CD38 under physiological conditions in naïve T cells maintains a quiescent state, while in pathological states, it is required for executing effector function upon antigenic stimulation partially by transducing the signal for T cell activation (Cho et al., 2000; Zaunders et al., 2005; Ghosh et al., 2023). CD39, encoded by *ENTPD1*, is an ectoenzyme that converts extracellular ATP to adenosine and has been implicated in Tregs and tumor-specific exhausted T effector cells (Borsellino et al., 2007; Canale et al., 2018; Simoni et al., 2018). It is noteworthy that in T_RM_, CD39 also works in concert with the other ectoenzyme CD73 to promote T_RM_ survival (Isaacs et al., 2024). Conversely, CD101, a T_RM_ marker for both CD4^+^ and CD8^+^ T cells (Kumar et al., 2017; Snyder et al., 2019), which inhibits T cell activation and IL-2 secretion (Soares et al., 1998), was upregulated in T_RM__1, but not in T_RM__2. Overall, these cellular features associated with differentially expressed cell surface markers between T_RM__1 and T_RM__2 reflect the phenotypic differences between these two T_RM_ subsets. T_RM__2 is in part immunophenotypically similar to tumor-infiltrating cytotoxic CD38^+^ CD39^+^ CD4^+^ T cells observed in B7-H3 (CD276) knockdown (KD) tumors (Liu et al., 2023) and to a subset of T cells abundant in the gut mucosa of patients with CD and celiac disease expressing high levels of HLA-DR, CD161, and CD38 or CD39 (Bai et al., 2014; Christophersen et al., 2019; Mitsialis et al., 2020). Indeed, CD4^+^ CD103^+^ T_RM_ were clearly segregated in their ability to secrete IFN-γ by the expression of HLA-DR (Fig. S1 E). Our results therefore suggest that combining CD4 and CD103 with some of these surface markers may allow for the isolation and enrichment of T_RM__2, with high purity, especially for disease-associated CD4^+^ T cells with strong inflammatory and cytotoxic properties.

**Figure 2.**
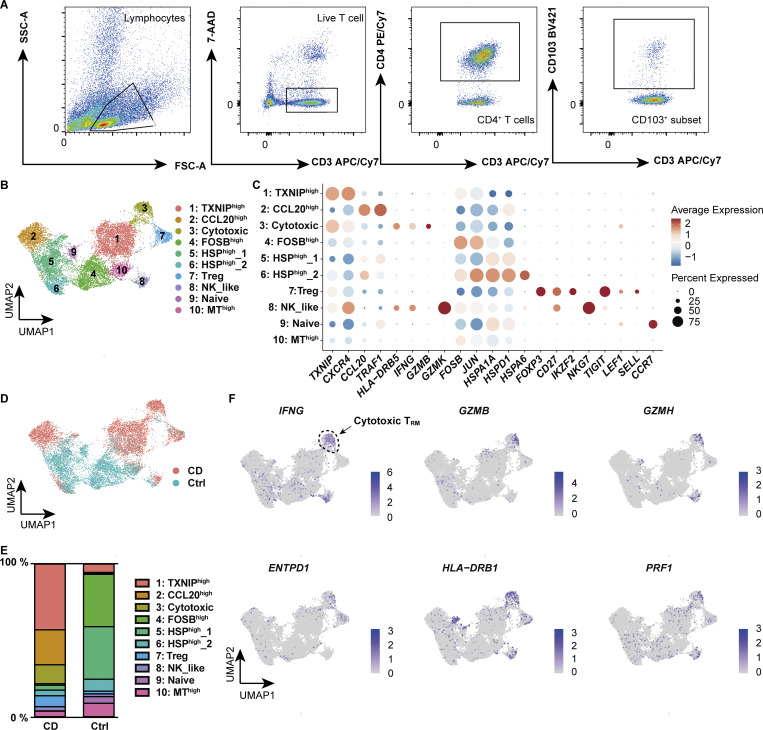
**scRNA-seq of CD4** ^**+**^ **T**_**RM**_ **in colon lamina propria. (A)** Gating strategy for the identification of CD4^+^ CD103^+^ T cells. **(B)** UMAP plot illustrating the annotation of lamina propria CD4^+^ CD103^+^ T cells derived from CD (*n* = 6, Patient 4–9 in Table S1) and control (*n* = 6, Ctrl-Patient 4–9 in Table S1) colon samples. **(C)** Expression levels of selected RNA markers across identified clusters. The dot size represents the percentage of cells expressing the gene within each cluster, while the color intensity reflects the average expression level. **(D)** Distribution of cells originating from CD (red) and control (blue) samples. **(E)** Proportional distribution of clusters across different disease conditions. Each bar represents 100% of the cells from a given disease, with segments indicating the relative abundance of each cluster. **(F)** Feature plot showcasing the expression of key marker genes characteristic of the cytotoxic cluster. The intensity of the color represents the expression levels of each gene, with higher expression indicated by darker shades.

**Figure S2.**
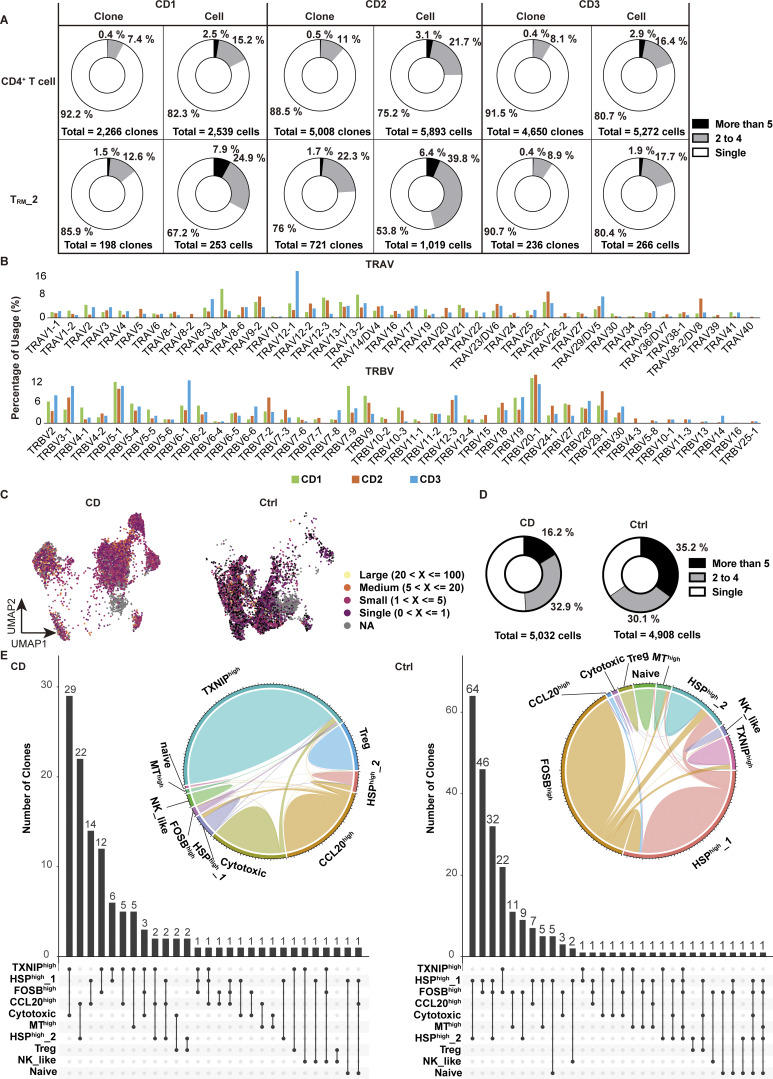
**TCR analysis of T**_**RM**_ **in colon lamina propria. (A)** Pie chart visualizing the distribution of TCR-sharing patterns among CD4^+^ T cells and T_RM__2 cells from each CD sample in CITE-seq data. The chart segments represent cells with TCRs shared by five or more cells, two to four cells, and TCRs unique to a single cell. Both the number of cells and clones are displayed. **(B)** Clonal usage data of TRAV and TRBV in T_RM__2 from the CITE-seq data. **(C)** UMAP plot illustrating the clone size of each cell in CD4^+^ CD103^+^ T cells from CD and control samples. **(D)** Pie chart visualizing the distribution of TCR-sharing patterns among CD4^+^ CD103^+^ T cells from CD and control samples. The chart segments represent cells with TCRs shared by five or more cells, two to four cells, and TCRs unique to a single cell. **(E)** Circos plot and UpSet plot demonstrating the overlap of unique individual TCR clonotypes between each cluster.

An independent scRNA-seq dataset of flow cytometry (FACS)–sorted CD4^+^ CD103^+^ T_RM,_ which allowed for more detailed profiling of T_RM_, further demonstrated the high diversity of T_RM_ (Fig. 2, A–C; and Table S2), with clear demarcation into CD-predominant and control-predominant subsets (Fig. 2, D and E). In this CD4^+^ CD103^+^ T_RM_ dataset, one of the CD-predominant subsets, which was assigned as cytotoxic T_RM_, was marked by the high expression of *IFNG*, *GZM*s, and *PRF1* (Fig. 2 F), resembling profiles observed during microbial infections (Glennie et al., 2015; Nguyen et al., 2023). Consistent with the findings on T_RM__2 in CITE-seq, *HLA-DRB1* was indeed highly and specifically expressed in the cytotoxic subset of the CD-dominant population (Fig. S2 F). These findings indicate that a certain subset of CD4^+^ CD103^+^ T_RM_ in the CD gut was programmed for effector- and innate-like functions.

### A combined analysis of the transcriptome and TCR repertoire reveals polyclonality of CD-specific T_RM_

**Figure 3.**
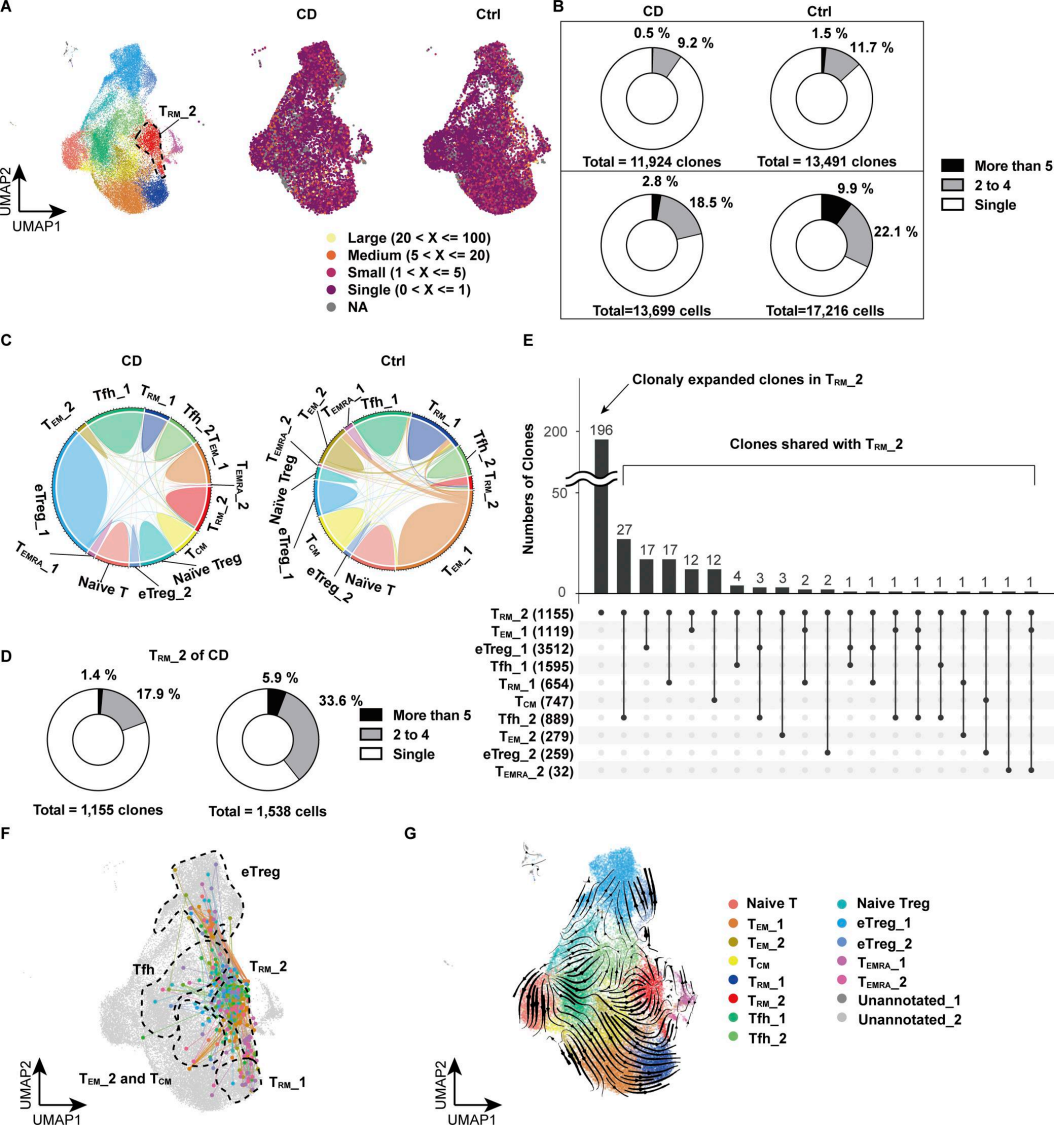
**Clonal sharing among CD4** ^**+**^ **T cells in the colon lamina propria. (A)** UMAP plot illustrating the clonal size of each cell from CD (*n* = 3, CD Patient 1–3 in Table S1) and control (*n* = 3, Ctrl-Patient 1–3 in Table S1) colon samples. **(B)** Pie chart visualizing the distribution of TCR-sharing patterns among all CD4^+^ T cells from CD and control samples. The chart segments represent cells with TCRs shared by five or more cells, two to four cells, and TCRs unique to a single cell. Both the number of clones (top) and cells (bottom) are displayed. **(C)** Circos plot demonstrating the overlap of unique individual TCR clonotypes between each cluster. **(D)** Pie chart visualizing the distribution of TCR-sharing patterns among all cells from the T_RM__2 cluster of CD samples. The chart segments represent cells with TCRs shared within T_RM__2 by five or more cells, two to four cells, and TCRs unique to a single cell. Both the number of clones (left) and cells (right) are displayed. **(E)** UpSet plot showing the distribution of TCR-sharing patterns among cells from the T_RM__2 cluster of CD samples, highlighting TCR overlaps between T_RM__2 and other clusters. The numbers in parentheses indicate the number of clones in each T cell subset. **(F)** UMAP plot showcasing cells that share TCRs with T_RM__2 cells from the CD samples. The dots represent cells outside of the T_RM__2 cluster that share the same TCR as cells within T_RM__2, along with the T_RM__2 cells that have matching TCRs. The lines connect cells with common TCRs. **(G)** UMAP plot with RNA velocity vectors overlaid on cells, indicating predicted future states and dynamic transitions in the transcriptional landscape.

We next explored whether cells comprising T_RM__2 respond to specific antigens to proliferate and share antigenicity with other T cell subsets. To this end, we performed single-cell TCR (scTCR) repertoire analysis with our CITE-seq dataset, which detected α-chain in 79.7%, β-chain in 88.5%, and paired αβ-chains in 78.5% of cells. Overall, gut CD4^+^ T cells of CD patients and controls were polyclonal (Fig. 3, A and B), with 9.7% and 13.2% of clonotypes shared by two or more cells, respectively (Fig. 3 B, upper panel). Additionally, 21.3% and 32% of CD- and control-derived CD4^+^ T cells, respectively, shared clones with other cells (Fig. 3 B, lower panel). Interestingly, there was slightly more clonal overlap between T_RM__2 and other CD4^+^ T cell subsets in control samples, possibly reflecting clonal proliferation of T cells in the peri-cancer region (albeit pathologically normal mucosa), supporting the previous findings that tumor-infiltrating lymphocyte clones are also clonally expanded in adjacent nontumorous tissues (Penter et al., 2019) (Fig. 3, A–C; and Fig. S2 A). Focusing on the clonotypes of T_RM__2 in CD patients, we found that 19.3% of clonotypes were shared by two or more cells within T_RM__2, while 39.5% of cells comprising T_RM__2 shared clones with other cells in T_RM__2, with no specific TRAV and TRBV usage (Fig. 3 D and Fig. S2, A and B). This aligns with findings in an independent CD4^+^ CD103^+^ T_RM_ dataset, where 49.1% of cells in CD patients and 65.3% in controls shared clones with at least two cells (Fig. S2, C and D). Furthermore, this CD4^+^ CD103^+^ T_RM_ dataset revealed that CD-predominant cytotoxic T_RM_ fraction shared the highest number of clones with the *TXNIP*^high^ T_RM_ subcluster, suggesting potential interconversion between CD-predominant subclusters (Fig. S2 E).

Since T cells sharing a pair of TCRαβ chains are presumed to be derived from a common ancestor, scTCR repertoire analysis combined with transcriptional characterization by scRNA-seq can provide information on lineage plasticity (Han et al., 2014; Stubbington et al., 2016; Pai and Satpathy, 2021). We therefore evaluated the distribution of clonotypes comprising T_RM__2 across CD4^+^ T cell subsets (Fig. 3, E and F). Most clonotypes were unique to T_RM__2 with minimal clonal overlap with other CD4^+^ T cell subsets in the lamina propria. In CD samples, 196 clonotypes comprising T_RM__2 were shared by at least two cells within the T_RM__2 subset, exceeding the overlap between other subsets (Fig. 3 E). The subcluster with the next highest clonal overlap with T_RM__2 was follicular helper T cell_2 (Tfh_2) with 27 clones, followed by 17 clones with T_RM__1 (Fig. 3, E and F). Considering the number of clones in each subset, clonotype overlap with T_RM__2 was highest for the following three subsets: 3.0% (27/889) for Tfh, 2.6% (17/654) for T_RM__1, and 1.6% (12/727) for central memory T cells (T_CM_). This aligns with previous reports showing potential plasticity of Tfh as revealed by epigenetic profiling of each T cell subset (Nakayamada et al., 2011; Lu et al., 2011; Oestreich et al., 2012). Additionally, epidermal T_RM_ clones have been shown to overlap with circulating T_CM_-like cells, suggesting that circulating T cells may serve as a source for T_RM_ (Zitti et al., 2023).

Our finding that the clonotypes comprising T_RM__2 are rarely distributed among other CD4^+^ T cell subsets may suggest that circulating T cells, rather than gut T cells, may acquire the potential to differentiate into T_RM__2 before tissue entry. To further complement the results of our TCR repertoire analyses, we applied RNA velocity to predict the direction of cell differentiation based on the dynamics of RNA transcription and splicing within cells. RNA velocity revealed trajectories going toward T_RM__2 from various subsets, such as Tfh_2, T_CM_, and T_RM__1, but none in the reverse direction was observed (Fig. 3 G). This suggests that T_RM__2 may differentiate from these subsets, regardless of whether the differentiation occurs during or after gut entry. Altogether, these results suggest that T_RM__2 may primarily derive from circulating T cells and partially from gut-resident T cells, likely induced by environmental niches during and after tissue entry, rather than by specific antigens.

### Imprinting of a subset of CD-specific T_RM_ occurs prior to tissue entry

**Figure 4.**
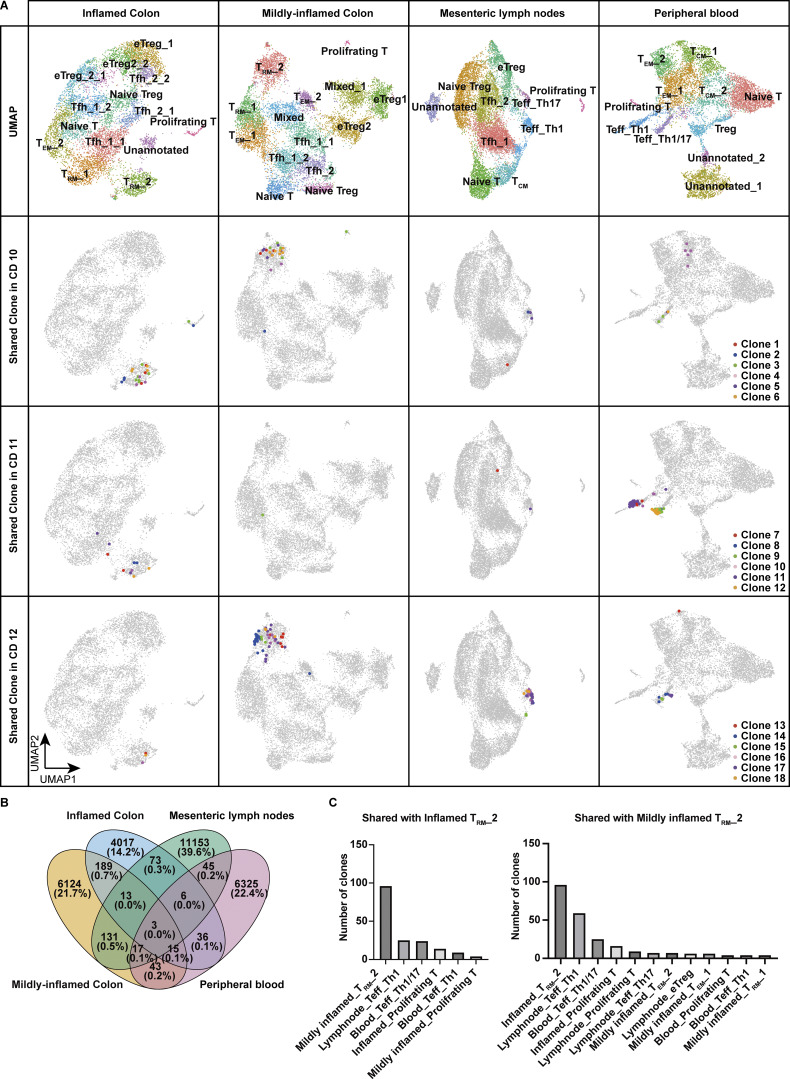
**Single-cell TCR repertoire analysis across blood, lymph node, and colon. (A)** Upper panels: UMAP plots showing the annotation of CD4^+^ T cells isolated from inflamed colon, mildly inflamed colon, MLN, and peripheral blood of CD patients (*n* = 3, CD Patient 10–12 in Table S1). Lower three panels: Top six CD4⁺ T cell clones within T_RM__2 shared across different tissues in each patient. **(B)** Venn diagram showing the total number of shared clones across tissues. **(C)** Number of clones shared with T_RM__2 of inflamed and mildly inflamed colon in each CD4^+^ T cell cluster. Clusters sharing more than five clones are displayed.

To investigate whether T_RM__2 originates from circulating T cells, we performed comparative scTCR repertoire analysis of CD4^+^ T cells isolated from the colon, mesenteric lymph nodes (MLN), and peripheral blood of three patients with CD who underwent surgical resection. 10,417 cells from mildly inflamed tissue, 7,514 cells from inflamed tissue, 12,644 cells from MLN, and 8,724 cells from peripheral blood were analyzed. Because completely uninflamed intestinal tissue was rarely available in CD surgeries, we analyzed both mildly and severely inflamed regions of the colon. Colonic CD4^+^ T cells were annotated using reference mapping with our current CITE-seq dataset, whereas CD4^+^ T cells from MLN and blood were annotated based on differentially expressed genes. T_RM__2 was detected in both mildly and severely inflamed colonic mucosa and represented the subset with the highest degree of clonal sharing across intestinal samples (Fig. 4, A and B). Clonotypes comprising T_RM__2 exhibited substantial overlap with those found in the Th1 subset, characterized by the expression of *TBX21* and *IFNG*, in the MLN, and with the Th1/Th17 subset, defined by the expression of *RORC* and *TBX21*, in the peripheral blood (Fig. 4, A and C). In contrast, minimal clonal overlap was observed between T_RM__2 and other CD4^+^ T cell subsets. These findings suggest that at least a subset of T_RM__2 originates from circulating Th1/Th17 cells, potentially differentiated from Th17 precursors. Therefore, it is likely that pro-inflammatory programming of T_RM__2 is initiated in the circulation or MLN before entering the gut and later reinforced by local signals that promote tissue residency and effector function.

### RUNX2 and BHLHE40 are selectively expressed in CD-associated CD4^+^ T_RM_

**Figure 5.**
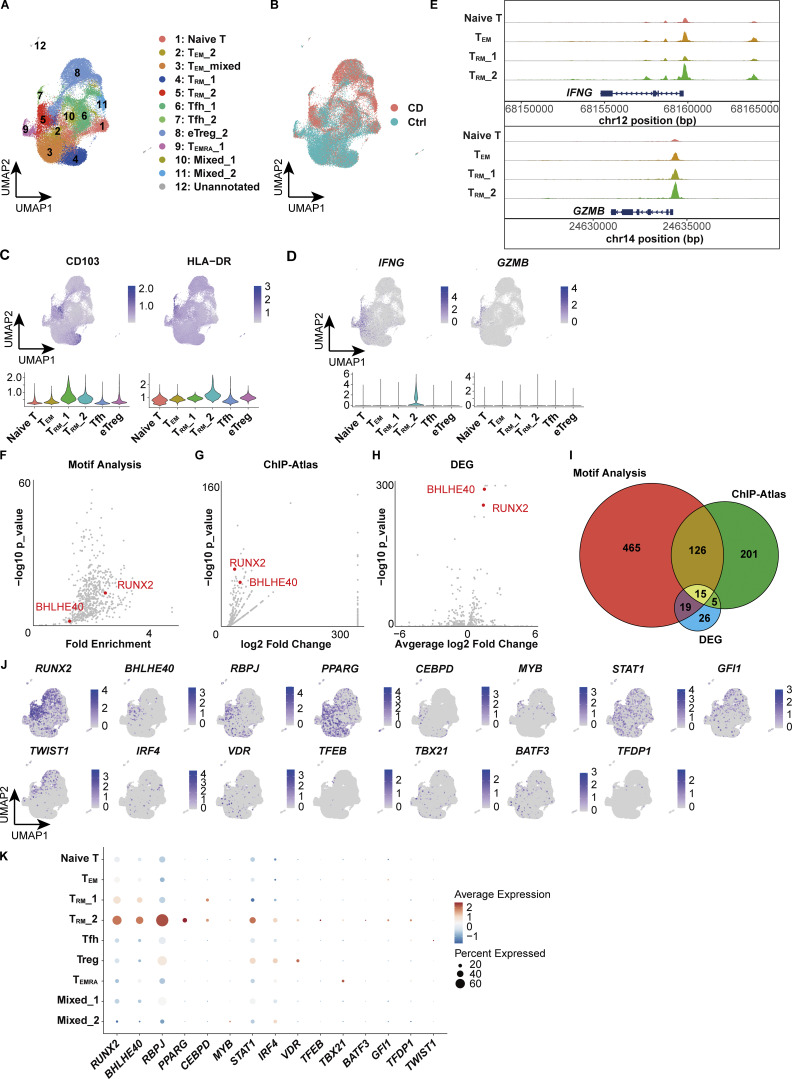
**scMultiome predicts transcriptional regulators of T**_**RM**_ **_2. (A)** UMAP plot depicting the annotation of colonic lamina propria CD4^+^ T cells derived from CD (*n* = 3, CD Patient 1–3 in Table S1) and control (*n* = 3, Ctrl-Patient 1-3 in Table S1) samples. The clusters were merged as they were assigned to the same cell population through reference mapping (Fig. S3 D). **(B)** Distribution of cells originating from CD (red) and control (blue) samples. **(C)** Feature plot illustrating the predicted expression of CD103 and HLA-DR as determined by the MapQuery function. **(D)** Feature plot showcasing the expression of *IFNG* and *GZMB*. **(E)** Coverage plot of the *IFNG* and *GZMB* loci. **(F)** Predicted TF binding within DARs of the T_RM__2 cluster. Differential accessibility was computed using Signac and Seurat, while TF binding was analyzed with motif analysis using Signac. **(G)** Predicted TF binding within DARs of the T_RM__2 cluster. TF binding was analyzed with ChIP-Atlas. **(H)** Volcano plot depicting differentially expressed TF-coding genes. *RUNX2* and *BHLHE40* are highlighted in F–H. **(I)** Venn diagram illustrating the number of TFs identified from the three analyses (F–H). **(J)** Expression patterns of 15 TFs identified from three analyses. **(K)** Expression levels of 15 TFs across clusters. The dot size indicates the percentage of cells expressing the gene within each cluster, while color intensity reflects the average expression level.

**Figure S3.**
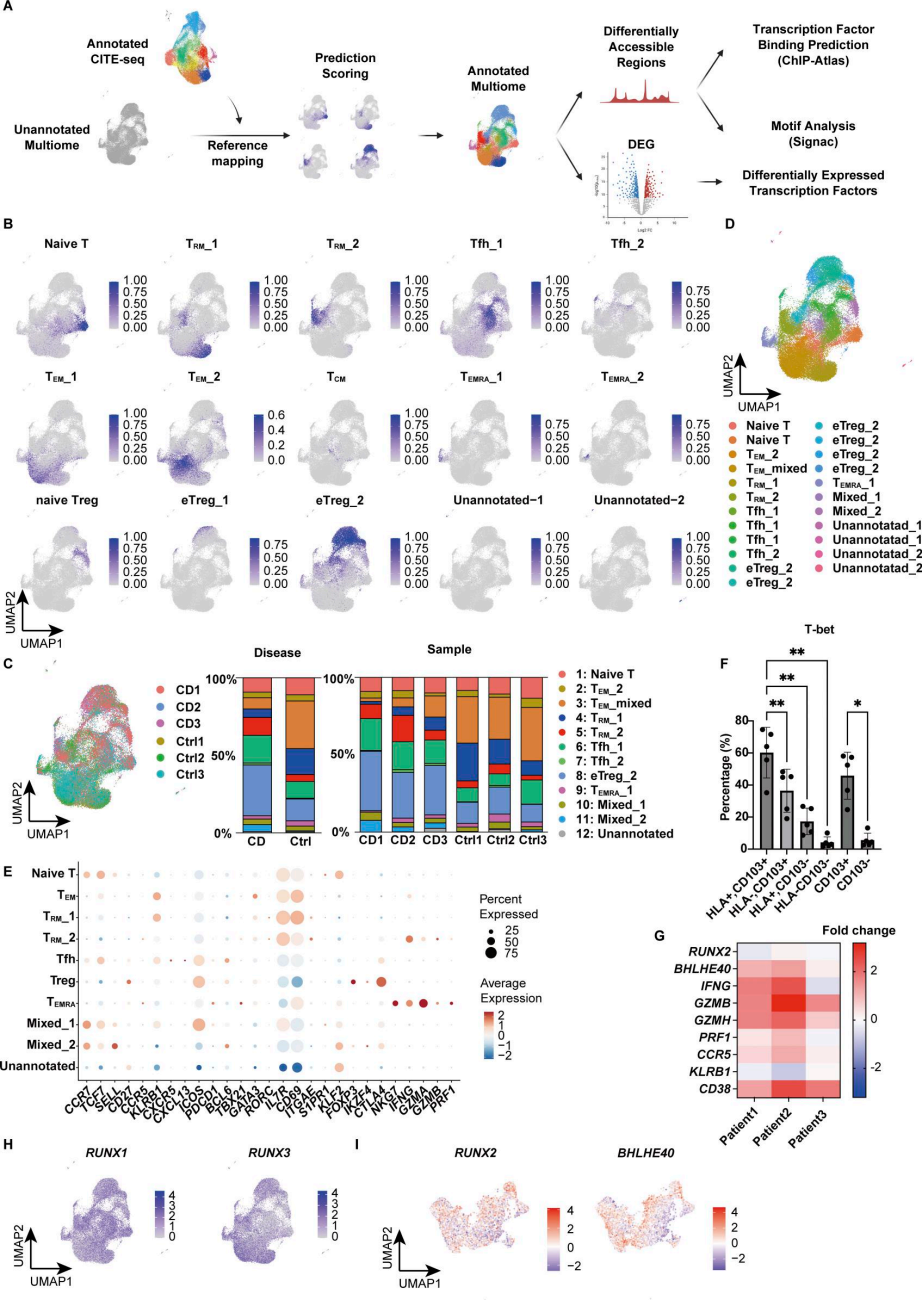
**Characterization of clusters in scMultiome data by reference mapping. (A)** Overview of reference mapping and TF prediction. **(B)** Feature plot displaying the prediction score calculated by the MapQuery function. **(C)** UMAP showing the proportion of each sample (CD: CD Patient 1–3; Control: Ctrl-Patient 1–3 in Table S1) and bar chart showing the proportion of disease and each sample. **(D)** UMAP plot showing the annotation of each cluster based on the prediction score from reference mapping. **(E)** Expression levels of selected RNA markers across identified clusters. The dot size represents the percentage of cells expressing the gene within each cluster, while the color intensity reflects the average expression level. **(F)** T-bet expression in each CD4^+^ T cell subset. Statistical significance for the comparisons was determined using RM one-way ANOVA, Dunnett’s multiple comparison test. *n* = 5 per group. *P < 0.05, **P < 0.01. **(G)** Heatmap showing the fold change in gene expression levels in HLA-DR^+^ T_RM_ cells relative to HLA-DR^−^ T_RM_ cells. **(H)** Feature plot of *RUNX1* and *RUNX3* expression. **(I)** TF activity inference calculated by the decoupleR package based on the DoRothEA network. Red indicates high activity, and blue indicates low activity.

**Figure 6.**
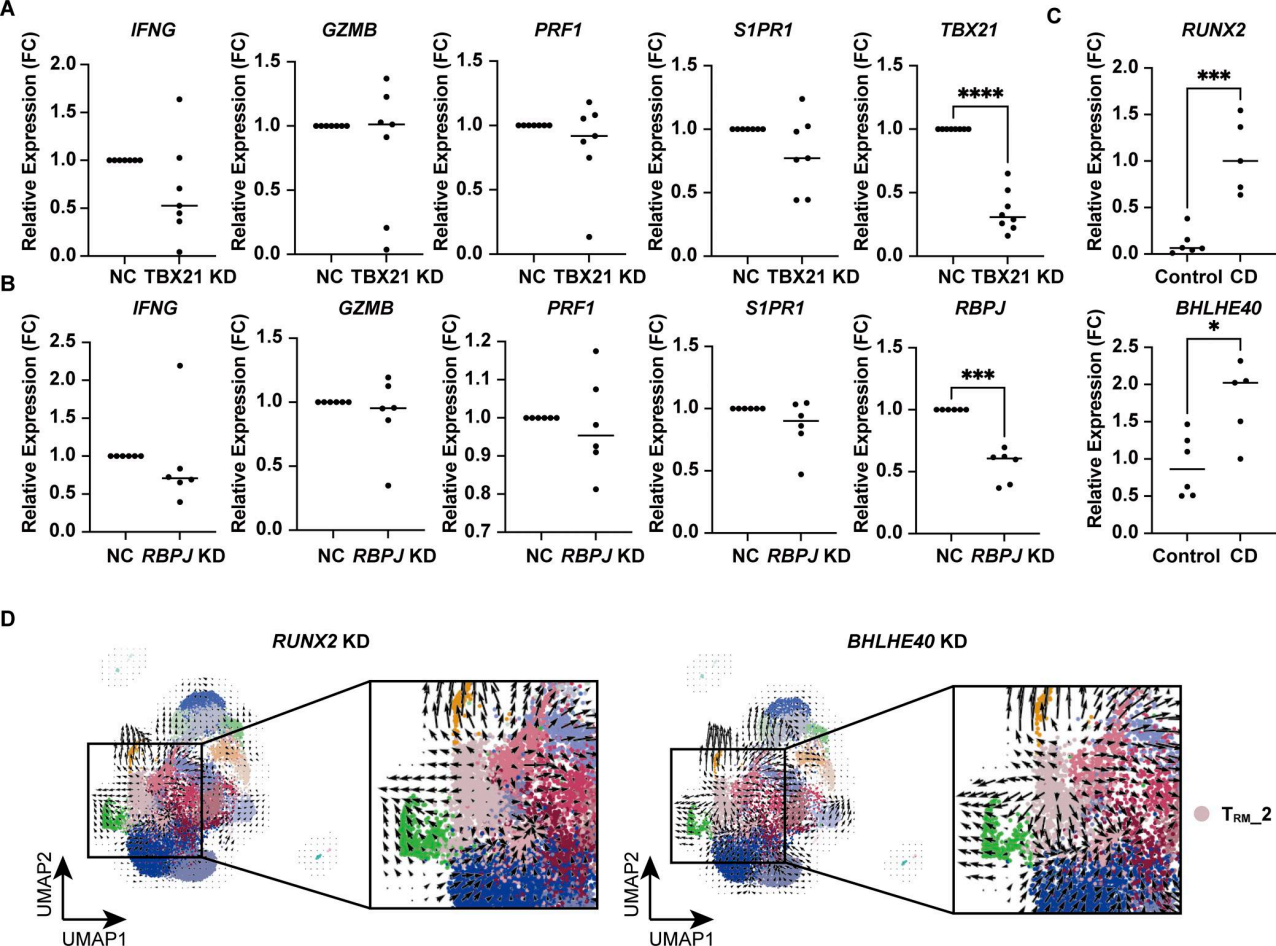
**Functional analysis of candidate TFs regulating T**_**RM**_ **_2. (A)** qPCR analysis of *TBX21* KD in colonic CD4^+^ T cells from CD patients. *n* = 7 per group. **(B)** qPCR analysis of *RBPJ* KD in colonic CD4^+^ T cells from CD patients. *n* = 6 per group. **(C)** Expression of *RUNX2* (upper panel) and *BHLHE40* (lower panel) in colonic CD4^+^ CD103^+^ T cells from control (*n* = 6) and CD (*n* = 5) samples by qPCR. Statistical significance for the comparisons for A–C was determined using a paired *t* test. *P < 0.05, ***P < 0.001, ****P < 0.0001. **(D)** CellOracle simulations of *RUNX2* (left) and *BHLHE40* (right) KD, showcasing cell-state transition vectors.

While gene expression analysis alone provides limited knowledge of the regulatory mechanisms underlying each T cell subset, complementing it with an understanding of the transcriptional regulatory circuits comprising TFs and their interacting DNA regulatory elements may reveal novel insights embedded in the regulatory code. We therefore leveraged the 10x Chromium Single Cell Multiome ATAC + Gene Expression assay to conduct paired analyses of the transcriptome and chromatin accessibility from the same cells. We profiled CD4^+^ T cells isolated from the colonic lamina propria of CD patients and controls (Fig. 1 A) and obtained joint gene expression and chromatin accessibility profiles from 42,553 cells comprising 18,284 cells for CD and 24,269 cells for control. We next projected a UMAP embedding based on both transcriptome and chromatin accessibility datasets using the Harmony algorithm (Korsunsky et al., 2019) and weighted nearest neighbor analysis (Fig. 5 A) (Hao et al., 2024). For proteins whose expression is mainly regulated by posttranscriptional or posttranslational modifications, gene expression levels do not necessarily correlate with those of proteins. For example, CITE-seq data and our previous report (Yokoi et al., 2023) showed that the expression of CD103, one of the crucial markers of human disease–specific CD4^+^ T_RM_, partially overlaps but does not coincide with the expression of its coding gene *ITGAE*, particularly in CD4^+^ T cells; CD103 is highly localized in T_RM_, while *ITGAE* is indeed highly expressed in T_RM_, but is also sporadically expressed in other subsets (Fig. 1, H and I). By applying multimodal reference mapping (Fig. S3 A) to our CITE-seq dataset and Multiome dataset, a cluster corresponding to each T cell subset in CITE-seq was assigned on the Multiome UMAP (Fig. 5 A and Fig. S3, B and D). This provided the resource data of gut T cells that combined transcriptome, proteome, and epigenomic information from the same CD patients into a single map. In congruence with our CITE-seq dataset, we identified a T_RM__2 cluster specifically enriched in CD but almost absent in control samples, whereas a cluster assigned as T_RM__1 was predominant in controls and poorly present in CD (Fig. 5 B and Fig. S3 C). Indeed, CD103 almost exclusively mapped to T_RM_s, and T_RM__2 expressed higher levels of HLA-DR, *IFNG*, and *GZMB*, compared with other CD4^+^ T cell subsets (Fig. 5, C and D). In addition, all T cell subsets exhibited transcriptional signatures characteristic of their annotated T cell subset as determined by reference mapping, validating successful data integration (Fig. S3 E). Consistent with high *IFNG* and *GZMs* expression in T_RM__2 (Fig. 5 D), the peak signals of open chromatin structures in their promoter regions were increased in T_RM__2 compared with those of naïve T, T_EM_s, and T_RM__1 (Fig. 5 E). To further identify gene regulatory programs that distinguish T_RM__2 from other CD4^+^ T cell subsets, TF motifs with differentially accessible regions (DARs) between T_RM__2 and the rest of CD4^+^ T cell subsets were identified using the ATAC modality of the single-cell Multiome (scMultiome) (Fig. 5 F). Subsequently, putative TFs that potentially bind to these DARs were ranked in order of P values by referring to the peak browser in ChIP-Atlas, a publicly accessible chromatin immunoprecipitation sequencing database (Zou et al., 2024) (Fig. 5 G). Since the number of T_RM__2-associated candidate TFs identified by each analysis was still large, we further considered differentially expressed TF-coding genes between T_RM__2 and other subsets from our scRNA-seq datasets (Fig. 5 H). Cross-referencing the results of these three analyses revealed 15 TFs as candidate molecules associated with T_RM__2 (Fig. 5, I–K). These TFs included *TBX21*, which encodes T-bet, the master regulator of Th1 differentiation. Indeed, flow cytometry analysis revealed that T-bet was expressed at higher levels in CD4^+^ CD103^+^ T_RM_, compared with CD4^+^ CD103^-^ T cells, and its expression was further elevated in cells expressing HLA-DR (Fig. S3 F). Although downregulation of T-bet has been reported to promote signaling pathways essential for CD8^+^ T_RM_ formation (Mackay et al., 2015; Evrard et al., 2022), the precise role of T-bet in CD4^+^ T_RM_ development remains less well defined. KD of *TBX21* in gut CD4^+^ T cells derived from CD patients resulted in a modest trend toward decreased *IFNG* expression; however, no statistically significant changes were observed in the expression levels of *IFNG*, *GZMB*, *PRF1*, and *S1PR1* (Fig. 6 A). These findings suggest that in addition to T-bet, other TFs may be involved in the regulation of IFN-γ production in CD4^+^ T_RM_ within the gut microenvironment of CD. Subsequent screening of 15 candidate TFs revealed that *RUNX2*, *BHLHE40*, *RBPJ*, and *PPARG* were highly expressed in T_RM__2 (Fig. 5 J). Among these four remaining TFs, *PPARG* was excluded as a candidate because it was expressed in <30% of T_RM__2 (Fig. 5, J and K). RBPJ, a central mediator of Notch signaling, has been reported to play a role in the formation or maintenance of CD4^+^ memory cells including T_RM_ in various tissues (Maekawa et al., 2015; Oja et al., 2018). KD of *RBPJ* in CD patient–derived gut CD4^+^ T cells did not affect the expression of *IFNG* or *S1PR1*, suggesting that *RBPJ* is unlikely to play a major role in the T_RM__2 phenotype, defined by both Th1-like and tissue retention properties (Fig. 6 B). Accordingly, RUNX2 and basic helix–loop–helix family member e40 (BHLHE40) emerged as the most likely candidates for regulating T_RM__2-specific transcriptional programs. Consistently, qPCR analysis confirmed that the expression of *RUNX2* and *BHLHE40* in CD103^+^ CD4^+^ T cells from the colon was significantly higher in patients with CD compared with controls (Fig. 6 C). Furthermore, isolated HLA-DR^+^ CD103^+^ CD4^+^ T cells exhibited the elevated expression of *BHLHE40*, as well as *IFNG* and *GZMB*, compared with the HLA-DR^−^ CD103^+^ CD4^+^ T cell population, supporting the notion that *BHLHE40* is more highly expressed in Th1-skewed T_RM_ compared with other T_RM_ subset (Fig. S3 G). Importantly, the expression of other RUNX family members, *RUNX1* and *RUNX3*, was not biased toward specific subsets within CD4^+^ T cells, and *RUNX3*, one of the key TFs of CD8^+^ T_RM_ induction, was highly expressed in T_RM__1 rather than T_RM__2 (Fig. S3 H). Furthermore, TF activity inference calculated by the decoupleR package indicated high activity of both RUNX2 and BHLHE40 in CD-predominant CD4^+^ T_RM_ subsets compared with control-predominant CD4^+^ T_RM_ subsets (Fig. S3 I). To further understand the role of RUNX2 and BHLHE40 in T_RM__2, we applied CellOracle, *in silico* gene perturbation analysis, which simulates shifts in cell identity associated with TF perturbation, providing a systematic understanding of the role of TFs in controlling cell fate decisions and cellular functions (Kamimoto et al., 2023). Applying our scMultiome dataset to CellOracle predicted that the loss of either RUNX2 or BHLHE40 prevents CD4^+^ T cell specialization into T_RM__2, predisposing them to differentiate into other T cell subsets (Fig. 6 D). Taken together, our analyses suggest that RUNX2 and BHLHE40 are potential regulators involved in the generation and maintenance of T_RM__2, warranting functional validation to elucidate their specific roles.

### RUNX2 and BHLHE40 contribute to the phenotypes of CD-associated CD4^+^ T_RM_

**Figure 7.**
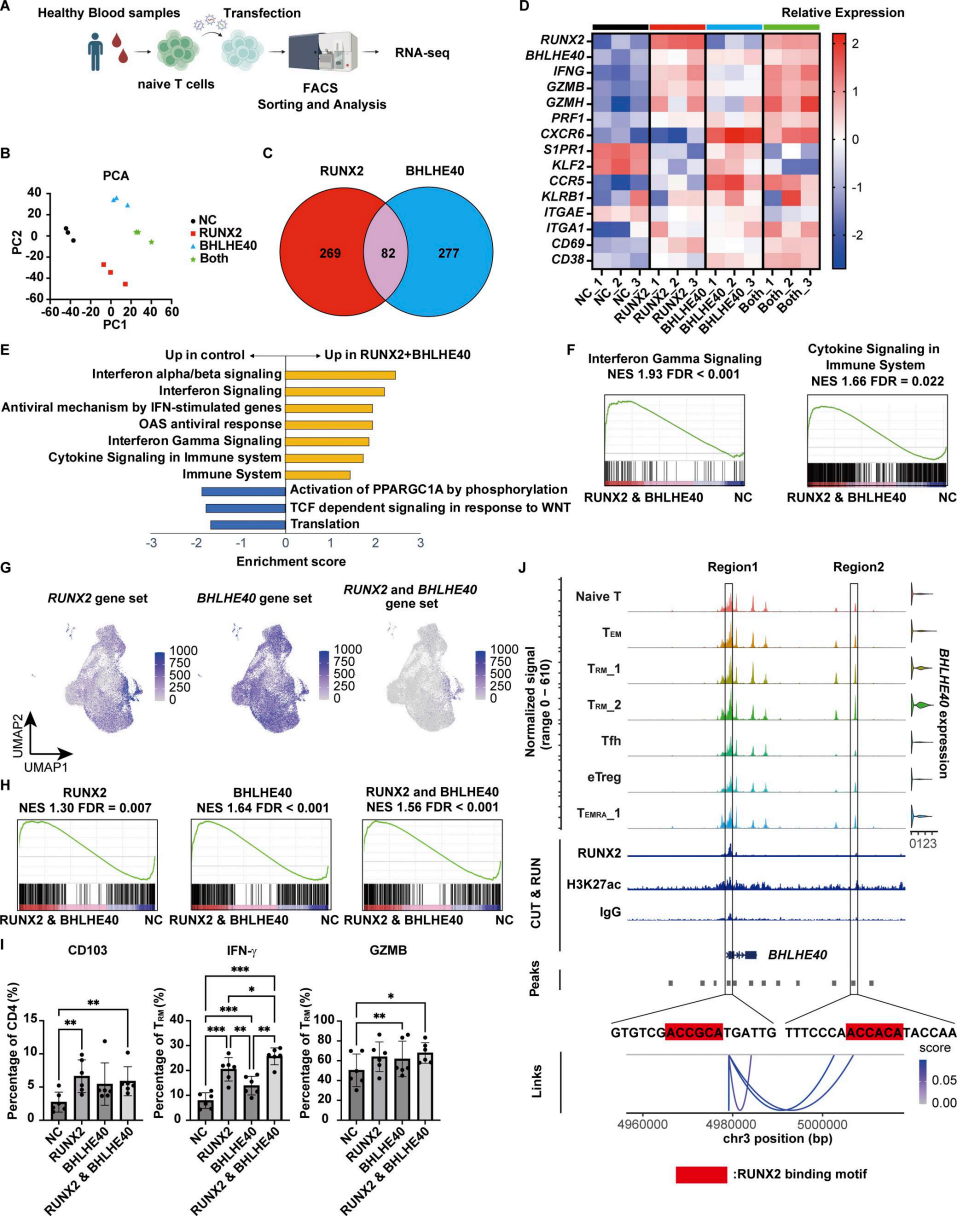
**RUNX2 and BHLHE40 expression confers T**_**RM**_ **_2 signature. (A)** Overview of the experimental steps. **(B)** PCA plot illustrating the variance in gene expression profiles across the four sample groups. Each point represents a sample, with its position reflecting the overall transcriptomic differences between groups. **(C)** Venn diagram showing the overlap number of upregulated genes (fold change > 2, P < 0.05). **(D)** Heatmap displaying the expression levels of selected genes across biological replicates for the following groups: control (NC), *RUNX2* overexpression (RUNX2), *BHLHE40* overexpression (BHLHE40), and *RUNX2* and *BHLHE40* overexpression (both). Rows represent individual genes, and columns represent samples, with color intensity indicating relative expression levels. *n* = 3 biological replicates. **(E)** Reactome pathway enrichment analysis was conducted on the differentially expressed genes identified in the *RUNX2* and *BHLHE40* overexpression groups. Significantly enriched pathways (FDR < 0.05) are shown in the bar chart. **(F)** GSEA was performed on the ranked list of genes from the differential expression analysis between *RUNX2* and *BHLHE40* overexpression samples and controls. The enrichment plot illustrates the distribution of genes from the Interferon Gamma Signaling and Cytokine Signaling in Immune System gene sets across the ranked gene list. The NES and FDR are provided. **(G)** Single-cell GSEA performed on the CITE-seq data (Fig. 1). Gene sets from the top 100 genes in *RUNX2*, *BHLHE40*, and *RUNX2* and *BHLHE40* overexpression were applied to the CITE-seq data. **(H)** GSEA of differentially expressed genes of T_RM__2 in CITE-seq data performed on *RUNX2*, *BHLHE40*, and both overexpression groups. **(I)** FACS analysis of CD103, IFN-γ, and GZMB in *RUNX2*- and *BHLHE40*-overexpressed T cells. *n* = 6 per group. Statistical significance for the comparisons was determined using RM one-way ANOVA, Dunnett’s multiple comparison test. *P < 0.05, **P < 0.01, ***P < 0.001. **(J)** Chromatin landscape at the *BHLHE40* locus as revealed by scMultiome and CUT&RUN peaks for RUNX2 and H3K27ac. Regions 1 and 2 indicate predicted RUNX2-binding peaks. Blue lines in the bottom represent the association between chromatin accessibility and gene expression. NES, normalized enrichment score.

**Figure S4.**
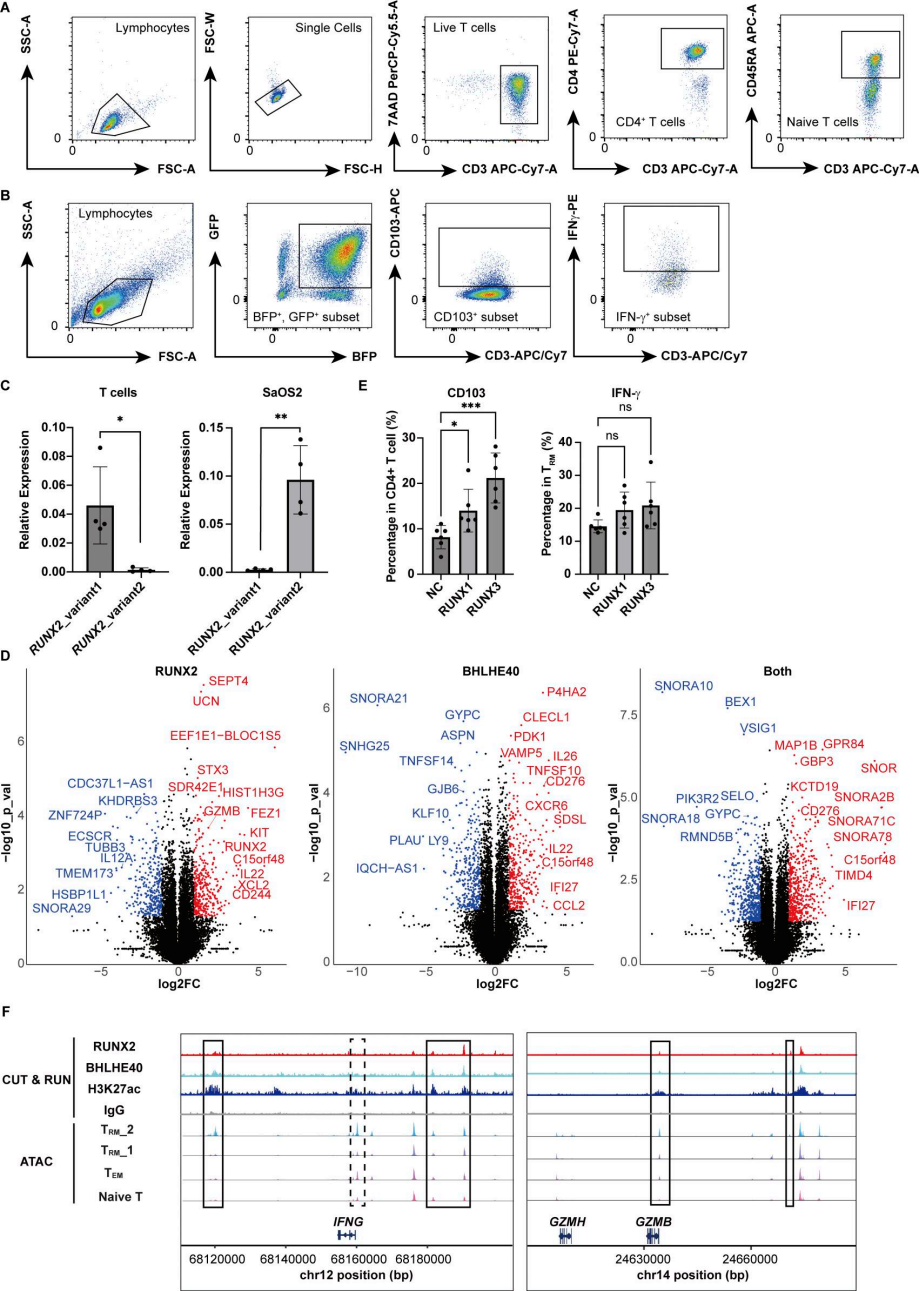
**Gating strategy of cell sorting, difference in RUNX2 expression by variants, and the effect of RUNX2 and BHLHE40. (A)** Gating strategy for the identification of CD4^+^ naïve T cells. **(B)** Gating strategy for the identification of GFP^+^ BFP^+^ CD4^+^ CD103^+^ T_RM_ cells and IFN-γ–positive T cells. **(C)** Bar graph showing the expression levels of *RUNX2* variant1 and variant2 in T cells and Saos2. *n* = 4 per group. Statistical significance for the comparisons was determined using a paired *t* test, *P < 0.05, **P < 0.01. **(D)** Volcano plot showing upregulated and downregulated genes in *RUNX2* overexpression (left), *BHLHE40* overexpression (center), and both overexpression groups (right). **(E)** FACS analysis of CD103 and IFN-γ in *RUNX1-* and *RUNX3*-overexpressed T cells. *n* = 5 per group. Statistical significance for the comparisons was determined using a paired *t* test and RM one-way ANOVA, Dunnett’s multiple comparison test, *P < 0.05, ***P < 0.001. **(F)** CUT&RUN peaks for RUNX2, BHLHE40, and H3K27ac, along with ATAC-seq peaks, are shown for T_RM__2, T_RM__1, T_EM_, and naïve T cells within the *IFNG* and *GZMB* regions. Solid boxes indicate open chromatin regions in T_RM__2 that colocalize with RUNX2 and BHLHE40 peaks, as detected by MACS2. The dashed box marks *IFNG* promoter regions without RUNX2 and BHLHE40 binding.

Given the TCR repertoire analysis suggesting that a considerable number of T_RM__2 are directly programmed and differentiated from blood-derived progenitors, *RUNX2* and *BHLHE40* were ectopically expressed in healthy donor–derived blood CD4^+^ T cells. Fluorescence-identified transfected T cells were subjected to bulk RNA-seq and FACS analysis to assess the effect of both TFs in CD4^+^ T cells (Fig. 7 A and Fig. S4, A and B). Since *RUNX2* is under control of distinct promoters, which give rise to two distinct *RUNX2* variants (Stock and Otto, 2005; Mevel et al., 2019), we selected the CD4-expressed *RUNX2* variant for both ectopic expression and subsequent *RUNX2* KD experiments; proximal promoter variant1 was expressed in gut-derived primary T cells from CD patients, while a human osteoblastoma cell line Saos2 barely expressed this variant but highly expressed distal promoter variant2 (Fig. S4 C). Principal component analysis (PCA) revealed that gene expression clearly separated the control and lentiviral overexpression groups for each TF (Fig. 7 B). Bulk RNA-seq analysis showed broad transcriptomic alterations recapitulating the T_RM__2 phenotype, with a substantial overlap of genes induced by the overexpression of RUNX2 and BHLHE40 (Fig. 7 C). For example, *IFNG*, cytolytic molecules such as *GZMs* and *PRF1*, and tissue-resident marker *CD69* were upregulated by *RUNX2* and *BHLHE40* induction, while tissue-egress markers *S1PR1* and *KLF2* were downregulated (Fig. 7 D and Fig. S4 D). Gene ontology analysis showed that significantly upregulated genes in the overexpression group were enriched in terms related to “Interferon Gamma Signaling” and “Cytokine Signaling in Immune System” (Fig. 7, E and F). Single-cell GSEA showed that the transcriptional signatures upregulated by the overexpression of either RUNX2 or BHLHE40 were distributed across various CD4^+^ T cell subsets. In contrast, T cells with a transcriptional signature induced by the overexpression of both TFs were highly enriched in T_RM__2 (Fig. 7 G). We next performed GSEA using the gene set significantly enriched in T_RM__2 from the CITE-seq dataset, and found that these genes were significantly associated with those upregulated by the overexpression of *RUNX2*, *BHLHE40*, and their combined overexpression (Fig. 7 H). Taken together, these results clearly showed that the induction of RUNX2 and BHLHE40 in CD4^+^ T cells drove the acquisition of T_RM__2 phenotypes. While *ITGAE* was not induced by either TF (Fig. 7 D), the overexpression of *RUNX2* variant1 resulted in the significant upregulation of the CD103 protein (Fig. 7 I). This is consistent with our previous findings that mRNA and protein levels of CD103 in CD4^+^ T cells do not completely correlate in humans. Additionally, IFN-γ secretion in CD4^+^ T_RM_ upon phorbol 12-myristate 13-acetate (PMA) and ionomycin stimulation was significantly upregulated by either RUNX2 or BHLHE40 expression, and further enhanced by the expression of both TFs. A similar trend was also observed in GZMB secretion (Fig. 7 I). To determine whether RUNX2 plays a unique role in this process or whether the overall dosage of RUNX family proteins is critical, we performed the overexpression of *RUNX1* and *RUNX3* transcripts driven by the distal promoter. CD103 was significantly induced by the overexpression of both *RUNX1* and *RUNX3*, whereas no observable effect on IFN-γ expression was noted (Fig. S4 E), suggesting the unique role of RUNX2.

Based on the substantial overlap of induced genes by *RUNX2* and *BHLHE40* overexpression (Fig. 7 C), and the significant induction of *BHLHE40* expression by RUNX2 overexpression (Fig. 7 D), we asked whether RUNX2 directly regulates BHLHE40. RUNX2-binding motifs were identified in the *BHLHE40* locus (Fig. 7 J). Chromatin profiling of human primary CD4^+^ T cells overexpressing RUNX2 revealed RUNX2 binding at two sites, coinciding with the presence of the activation-associated histone mark H3 lysine 27 acetylation. This suggests that RUNX2 controls the transcription of *BHLHE40*. Additionally, RUNX2 and BHLHE40 were predominantly recruited in T_RM__2 to the distal regions located 21,366 and 30,244 bp upstream, as well as 40,055 bp downstream from the transcription start site (TSS) of *IFNG*, and to the promoter region of *GZMB* (Fig. S4 F). This implicates a direct regulatory role of RUNX2 and BHLHE40 in the expression of these genes. However, considering that the ATAC-seq peak in the distal regions of *IFNG* has also been observed in other T cell subsets, and that there is no direct binding to the promoter region of *IFNG*, it is likely that the transcriptional control of *IFNG* by RUNX2 and BHLHE40 primarily occurs through indirect regulatory mechanisms, with minimal influence of direct binding.

**Figure 8.**
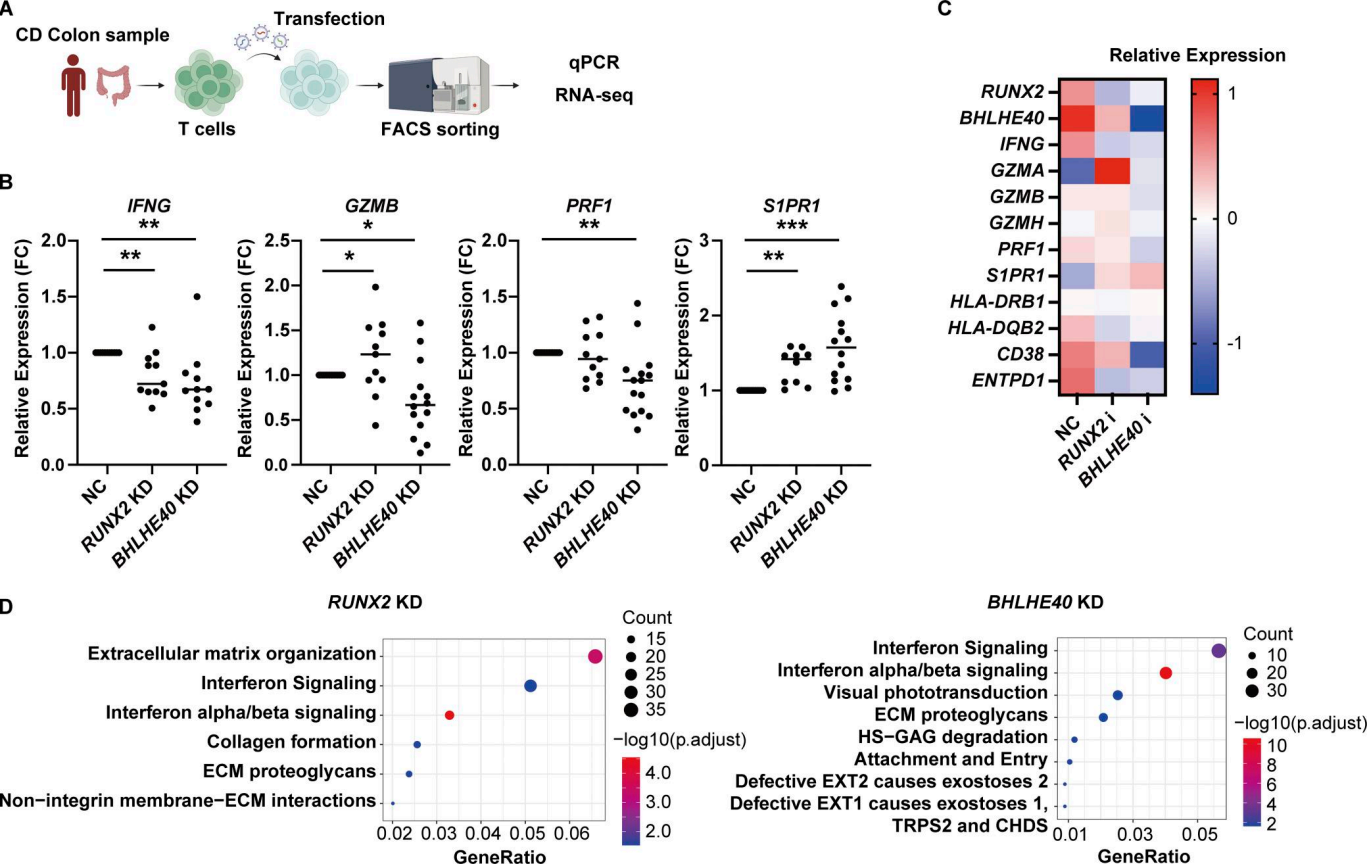
**KD of *RUNX2* and *BHLHE40* in colonic CD4** ^**+**^ **T cells in CD patients leads to a loss of functionality in T**_**RM**_ **_2. (A)** Overview of the experimental steps. **(B)** qPCR analysis of *RUNX2* and *BHLHE40* KD in colonic CD4^+^ T cells from CD patients. Statistical significance for the comparisons was determined using RM one-way ANOVA, Dunnett’s multiple comparison test. *n* = 11–15 per group. *P < 0.05, **P < 0.01, ***P < 0.001. **(C)** Heatmap showing the expression levels of selected genes across the following groups: control (NC), *RUNX2* KD (*RUNX2*i), and *BHLHE40* KD (*BHLHE40*i). Each row represents an individual gene, and each column represents a sample. Color intensity indicates the relative expression level. **(D)** Pathway analysis of downregulated genes from *RUNX2* KD (left) and B*HLHE40* KD (right).

**Figure S5.**
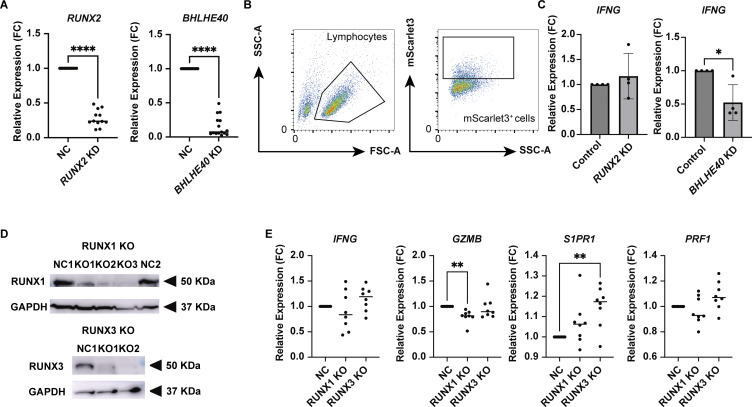
**RUNX2 and BHLHE40 KD, and RUNX1 and RUNX3 KO by the lentiviral CRISPR system. (A)** qPCR analysis of *RUNX2* and *BHLHE40* KD in CD patient–derived CD4^+^ T cells. *n* = 11–15 per group; statistical significance was determined using a paired *t* test, ****P < 0.0001. **(B)** Gating strategy of mScarlet3-positive infected cells indicating successful transfection. **(C)** qPCR analysis of *RUNX2* and *BHLHE40* KD T cells from control patient–derived CD4^+^ T cells. *n* = 4 per group; statistical significance was determined using a paired *t* test, *P < 0.05. **(D)** Validation of RUNX1 and RUNX3 KO by western blot. KO efficiency of RUNX1 and RUNX3 using three independent RNAs for RUNX1 (KO1–3), and two for RUNX3 (KO 1 and 2). KO2 for both RUNX1 and RUNX3 KO was used for the analysis. **(E)** qPCR analysis of *RUNX1* and *RUNX3* KO in colonic CD4^+^ T cells from CD patients. Statistical significance for the comparisons was determined using RM one-way ANOVA, Dunnett’s multiple comparison test. *n* = 8 per group. *P < 0.05, **P < 0.01, ****P < 0.0001. Source data are available for this figure: SourceData FS5.

We next asked whether downregulation of both TFs in patient-derived colonic T cells could reverse the inflammatory and tissue-resident phenotype of these cells (Fig. 8 A). A lentiviral CRISPRi system applied to CD4^+^ T cells isolated from the colon lamina propria of CD patients showed significant downregulation of *RUNX2* and *BHLHE40* expression (Fig. S5, A and B). *RUNX2* and *BHLHE40* KD significantly decreased PMA/ionomycin-induced *IFNG* expression (Fig. 8 B). This contrasts with the lack of *IFNG* suppression observed following *RUNX2* KD in the control samples, which reflects the very low expression of *RUNX2* in control CD4^+^ T cells (Fig. S5 C). In contrast, KD of *BHLHE40*—which is present at a certain basal level of expression in controls—resulted in a trend toward reduced *IFNG* induction upon PMA/ionomycin stimulation. Additionally, *GZMB* and *PRF1* were also downregulated by *BHLHE40* KD, suggesting that BHLHE40 has a stronger effect on the secretion of these cytotoxic molecules. Conversely, *S1PR1* expression was upregulated by the reduction of both TFs. Bulk RNA-seq analysis showed that KD of *RUNX2* or *BHLHE40* resulted in a reduction of coding genes for cell surface markers such as MHC class II genes, *CD38* and *ENTPD1*, which have been found to be potent T_RM__2 markers by CITE-seq (Fig. 8 C). Pathway analysis indicated a marked reduction of IFN signaling pathways in both groups (Fig. 8 D). On the other hand, CRISPR-mediated knockout (KO) of *RUNX1* and *RUNX3* in gut CD4^+^ T cells from CD patients did not result in significant changes in *IFNG* expression. In contrast, *RUNX3* KO led to a significant upregulation of *S1PR1* expression (Fig. S5, D and E). Together with the results from the *RUNX1* and *RUNX3* overexpression experiments, tissue residency appears to be regulated by shared functions among RUNX family members. In contrast, with respect to the Th1 properties of T_RM__2, RUNX2 may exert a distinct, nonredundant function that is not compensated by other RUNX family members such as RUNX1 or RUNX3.

These results indicate that both RUNX2 and BHLHE40 expressed in the inflamed gut contribute to shaping the cellular properties of pathologically relevant CD4^+^ T cells. Altogether, we propose that RUNX2 and BHLHE40 are key molecules for the acquisition of the cellular properties of disease-specific CD4^+^ T cells, such as Th1, and cytolytic propensity with tissue residency.

## Discussion

Various animal models of experimental colitis recapitulate certain aspects of IBD and have been widely used to investigate the molecular mechanisms underlying the disease. However, analyzing human samples from sites of inflammation is indispensable for a comprehensive understanding of the complex pathophysiology of IBD, as it is a multifactorial disease influenced by both genetic and environmental factors, and interspecies differences in the immune system cannot be ignored.

In this study, we analyzed human samples to elucidate the regulatory mechanisms of CD4^+^ T_RM_, particularly those associated with disease. The intestinal tract, constantly exposed to foreign substances, such as microorganisms and dietary components, can be the site of recurring inflammation. Therefore, it is reasonable to assume that dysregulation of the T_RM_ function, particularly immunological recall and the ability to initiate local immune responses, may contribute to the inflammation characteristic of IBD. In contrast to CD8^+^ T_RM_, which have been extensively studied, the developmental origins and transcriptional regulatory mechanisms of CD4^+^ T_RM_ remain largely unknown. The functional diversity of CD4^+^ T cells is dependent on key TFs that specify particular T cell lineages. By taking full advantage of multilayered single-cell analytic approaches, we provided the resource data of gut T cells that integrated epigenome, transcriptome, proteome, and TCR information from the same CD patients, which allow for the detailed characterization of CD-associated CD4^+^ T_RM_. We showed that both RUNX2 and BHLHE40 induced the CD-related T_RM_ phenotype, which was more robustly enhanced by the co-expression of the two TFs.

Our results demonstrate that BHLHE40 is involved not only in the induction of Th1 phenotype but also in the tissue retention properties of CD4^+^ T_RM_ by downregulating the tissue-egress markers. BHLHE40 is a member of the helix–loop–helix TFs that are expressed in a wide range of cell types and tissues, which acts as a transcriptional repressor by recruiting histone deacetylases to its target genes (Sun and Taneja, 2000) and outcompeting other transcriptional activators (Azmi et al., 2003). It also acts as a transcriptional activator through direct interactions with partner TFs or indirect effects mediated by other biological pathways (Kanda et al., 2016; Huynh et al., 2018). In mice, Bhlhe40 binds directly to the *Il10* locus and represses its transcription (Huynh et al., 2018; Yu et al., 2018), leading to decreased IFN-γ secretion from immune cells. It also binds to the *Ifng* locus distal from the TSS in T cells but not in myeloid cells (Huynh et al., 2018). This suggests that the transcriptional regulation of *Ifng* by Bhlhe40 is the consequence of either indirect regulation through repression of IL-10 signaling or T cell–specific direct activation. In natural killer T cells, Bhlhe40 serves as a cofactor to induce IFN-γ expression by interacting with T-bet (Kanda et al., 2016), a master regulator of Th1 differentiation (Szabo et al., 2000). In our study in human T cells, BHLHE40 binds directly to distal regions of *IFNG* with relatively low transcriptional activity, suggesting that BHLHE40 is mainly responsible for the induction of *IFNG* through indirect action. Additionally, Bhlhe40-deficient CD4^+^ T cells fail to induce colitis in a T cell transfer colitis model (Yu et al., 2018), further supporting our finding that induction of BHLHE40 expression in pathologically relevant CD4^+^ T cells in the gut is pathogenic to colitis. In contrast to its association with inflammation, the involvement of Bhlhe40 in tissue retention has been less described, with the exception to its control of CD8^+^ T_RM_ by mitochondrial metabolism programming (Li et al., 2019). Bhlhe40 is also required to maintain cell survival in cancer cells and lung-resident helper T cells that exhibit both Tfh and T_RM_ characteristics (Sethuraman et al., 2018; Son et al., 2021). The present study adds a mechanism by which BHLHE40 contributes to tissue residency; BHLHE40 represses tissue-egress marker *S1PR1*. In this study, we showed that *BHLHE40* expression is induced by RUNX2 overexpression, but its multifactorial regulation by additional factors beyond RUNX2 (Miyazaki et al., 2002; Butler et al., 2004; Lin et al., 2016), may underlie the limited overlap between RUNX2- and BHLHE40-regulated genes, possibly reflecting gene-specific thresholds of BHLHE40 activity.

The RUNX family of proteins, consisting of RUNX1, RUNX2, and RUNX3, contains a highly conserved Runt domain, which is responsible for DNA binding and interaction with the core-binding factor beta subunit. However, each RUNX protein plays distinct roles in various cell lineages and biological processes (Blyth et al., 2005; Ito et al., 2015). It is noteworthy that RUNX3 and RUNX1 are essential for the differentiation of CD8^+^ T cells and critical for the functionality of cytotoxic T lymphocytes (CTL) by repressing *CD4* and *Thpok* expression and supporting CD8 lineage commitment (Taniuchi et al., 2002; Woolf et al., 2003; Setoguchi et al., 2008). Unlike other tissues, ThPOK expression in CD4^+^ T cells from the gut is unstable, and a RUNX3-dependent ThPOK decrease causes CD4^+^ T cells to change from a helper to a CTL-like phenotype (Mucida et al., 2013; Reis et al., 2013). These MHC class II–dependent CTL are reported to be inactive at steady state even in the presence of antigen, but strongly activated under inflammatory conditions (Mucida et al., 2013). This is similar to our previous observation on the properties of CD-specific CD4^+^ T_RM_, which are strongly activated in the presence of common gamma-chain cytokines (Yokoi et al., 2023). Furthermore, RUNX3, whose expression is upregulated in a T-bet–dependent manner in CD4^+^ T cells under Th1-polarizing conditions, induces *Ifng* activation by working in cooperation with T-bet (Djuretic et al., 2007). Meanwhile, RUNX2 is a master regulator of bone development and osteoblast differentiation (Ducy et al., 1997; Komori et al., 1997; Otto et al., 1997), with limited reports in tissues and cells other than bones. RUNX2 transcriptional variants arise from alternative splicing and the use of different promoters, leading to the production of different isoforms of the RUNX2 protein (Stock and Otto, 2005; Wahlen et al., 2022; Mevel et al., 2019). Of these, inflammatory T cells express high levels of isoforms different from those predominantly expressed in the bone. In human CD4^+^ T cells, *RUNX3* was expressed across subsets, but was poorly expressed in T_RM__2. While *RUNX2* was primarily expressed in T_RM__2, the mechanisms by which *RUNX2* is induced in CD4^+^ T cells upon inflammation had remained unclear. Our overexpression and inhibition experiments of RUNX proteins in human CD4^+^ T cells suggest that both the dosage and the unique functional properties of RUNX2 are critical, particularly in driving the inflammatory T_RM__2 phenotype in the gut of CD patients.

There are three prevailing hypotheses regarding the origin of T_RM_: (1) a subset of circulating T cells possesses a superior potential for T_RM_ commitment prior to peripheral tissue entry, (2) differentiation into T_RM_ is induced after tissue entry in response to local cues, or (3) both (Kok et al., 2022). Our scTCR repertoire analyses revealed that clonotypes comprising T_RM__2 were mostly confined within the same subsets, with minor distribution among other CD4^+^ T cell subsets. This result may suggest that certain subsets of circulating T cells may be predisposed to differentiate into T_RM_ before tissue entry, consistent with previous reports (Matos et al., 2022; Zitti et al., 2023; Nguyen et al., 2023). Indeed, subsequent scTCR repertoire analysis combined with transcriptome profiling revealed signatures of intestinal T_RM_ precursors within the Th1/17-to-Th1 transition populations in the peripheral blood and MLN. On the other hand, minor clonal overlap with Tfh, T_RM__1, and T_CM_ was observed in some T_RM__2 in the gut. This is in line with the previous findings that CD4^+^ T_RM_ may arise from or in parallel with Tfh, with gene expression patterns associated with memory cell and T cell survival (Choi et al., 2013; Swarnalekha et al., 2021; Son et al., 2021), and that CD4^+^ T_RM_ require some aspects of the memory/Tfh program for long-term maintenance and survival (Nguyen et al., 2023).

In summary, we found that the dual expression of RUNX2 and BHLHE40 in the putative progenitor cell population induced a Th1-skewed T_RM_ phenotype, and most importantly, suppression of both TFs in patient-derived gut T cells mitigated these phenotypes. The present study provides mechanistic insights into the induction of pathogenesis-associated T cells in CD. However, the mechanisms underlying the induction of these TFs require further investigation. Elucidating these mechanisms will pave the way for potential therapeutic strategies targeting tissue-specific immune responses in CD.

## Materials and methods

### Preparation of CD4^+^ T cells from the peripheral blood

Peripheral blood mononuclear cells (PBMCs) were isolated using Ficoll-Paque PREMIUM 1.084 (Cytiva) according to the manufacturer’s protocol. Briefly, 10 ml of peripheral blood was diluted with an equal volume of phosphate-buffered saline (PBS). The diluted blood sample was layered onto Ficoll-Paque medium solution, followed by centrifugation at 400 *g* for 30 min at room temperature (RT). After removing the upper layer, the mononuclear cell layer was collected in a new tube and washed twice with FACS buffer (PBS supplemented with 2% fetal bovine serum albumin [FBS]). Isolated PBMCs were then used for CD4^+^ T cell isolation with CD4^+^ T Cell Isolation Kit (Miltenyi Biotec) according to the manufacturer’s protocol. In brief, PBMCs were resuspended in 40 μl of FACS buffer per 10^7^ total cells. 10 μl of CD4^+^ T Cell Biotin-Antibody Cocktail was added and incubated at 4°C for 5 min. After adding 30 μl FACS buffer, 20 μl of CD4^+^ T Cell MicroBeads Cocktail was added and incubated at 4°C for 10 min. Cells were applied to the prewashed LS column, and the flow-through containing CD4^+^ T cells was collected.

### Isolation of mononuclear cells from the intestinal lamina propria

Normal colonic mucosa was obtained from macroscopically unaffected areas of patients undergoing surgery for colorectal cancer. Inflamed colonic mucosa was obtained from surgically resected specimens of patients with CD. Briefly, colonic epithelial cells were dissociated by shaking in 5 mM ethylenediaminetetraacetic acid (EDTA) in Hanks’ balanced salt solution, followed by removal of the muscle layer. The mucosal layer was cut into pieces and digested with 1 mg/ml collagenase type I (Sigma-Aldrich) mixed with 0.07 IU/ml DNase in RPMI 1640 for 30 min at 37°C. Cells were centrifuged at 800 *g*, dispersed in EDTA solution, and washed in PBS. Lamina propria mononuclear cells (LPMC) were freshly used or frozen in liquid nitrogen until CD4^+^ T cell isolation or flow cytometry analysis.

### Isolation of mononuclear cells from the MLN

MLN were isolated as previously described (Roider et al., 2021). Briefly, MLN were excised from the mesentery and the surrounding adipose tissue was thoroughly removed. The isolated lymph nodes were transferred into a dish containing medium (RPMI 1640 supplemented with 10% FBS, 100 U/ml penicillin, 100 µg/ml streptomycin sulfate [Thermo Fisher Scientific]) and mechanically dissociated by mincing with scissors. The resulting tissue fragments were resuspended in medium and vigorously pipetted to release immune cells. The cell suspension was filtered through a 100-µm cell strainer. Additional medium was repeatedly added to the tissue fragments remaining on the strainer. The collected cells were centrifuged at 400 *g* for 5 min at 4°C, and the supernatant was carefully removed. Red blood cells were lysed using ACK lysis buffer, followed by another centrifugation at 400 *g* for 5 min at 4°C. After removing the supernatant, the cell pellet was resuspended in fresh medium and filtered through a 50-µm cell strainer, and then, the medium was repeatedly added to the residual fragments on the strainer. The cells were then centrifuged at 400 *g* for 5 min at 4°C and used for CD4^+^ T cell isolation.

### CD4^+^ T cell isolation from the intestinal lamina propria and MLN

Peripheral blood–derived CD4^+^ T cells, isolated LPMC, and mononuclear cells from MLN were stained with antibodies against CD3-APC/Cy7 (#300426; BioLegend) and CD4-PE/Cy7 (#557852; BD Biosciences) for 30 min at 4°C and washed two times with FACS buffer. For dead cell staining, cells were resuspended with FACS buffer containing 7-aminoactinomycin D (7AAD; #420404; BioLegend) and acquired on BD FACSMelody (BD Biosciences). CD3^+^ CD4^+^ 7AAD^−^ T cells were used for CITE-seq and scMultiome.

### Flow cytometry analysis

Colonic LPMC were stimulated with PMA (10 ng/ml), ionomycin (250 ng/ml), and monensin (GolgiStop; #554724; BD Pharmingen), and incubated at 37°C for 3 h. After stimulation, cells were harvested and washed with FACS buffer twice, followed by staining with antibodies against cell surface markers: CD3-APC/Cy7 (#300426; BioLegend), CD4-PE/Cy7 (#557852; BD Pharmingen), CD103-BV421 (#350214; BioLegend), and HLA-DR-APC (#559666; BD Pharmingen) for 30 min at 4°C. Cells were then washed with FACS buffer, treated with Cytofix/Cytoperm (BD Biosciences) for 20 min at 4°C, washed with perm/wash buffer, and centrifuged at 800 *g*. IFN-γ-FITC (#552887; BD Pharmingen) and T-bet-PerCP/Cy5.5 (#644805; BioLegend) dissolved in perm/wash buffer were added to the pellet and incubated for 30 min at 4°C, and then washed with perm/wash buffer. Cells were acquired on BD FACSCanto. Flow Cytometry Standard files were uploaded to FlowJo software and analyzed.

### scRNA-seq library preparation for lamina propria CD103^+^ CD4^+^ T cell data

Frozen LPMC were thawed, incubated with cell surface markers for 30 min at 4°C, and then subjected to dead cell staining. CD4^+^ CD103^+^ 7AAD^−^ cells were sorted on a BD FACSAria. After cell sorting, cells were incubated with Human TruStain FcX (#422301; BioLegend) for 10 min at 4°C, labeled with TotalSeq-C anti-human Hashtag antibody (Hashtag 1 to 6; #394661, #394663, #394665, #394667, #394669, #394671; BioLegend) for 30 min at 4°C, and resuspended in PBS with 0.04% bovine serum albumin (BSA) at a density of 1,000 cells/μl. Single-cell suspensions were processed through the 10x Genomics Chromium Controller following the protocol outlined in the Chromium Single Cell 5′ Reagent Kits v2 User Guide. For V(D)J repertoire profiling, full-length V(D)J regions were enriched from cDNA by PCR amplification with primers specific to the TCR constant regions by Human TCR Amplification Kit (10x Genomics). Enriched V(D)J segments were used for V(D)J-library construction. For cell surface protein analysis and cell hashing, feature barcode libraries were constructed from antibody-derived tag (ADT)/HTO-derived cDNA. Libraries were then sequenced on NovaSeq 6000 System (Illumina) in paired-end mode (read1: 26 bp; read2: 91 bp). The resulting raw reads were processed using Cell Ranger (10x Genomics).

### CITE-seq library preparation for lamina propria CD4^+^ T cell data

Freshly isolated LPMC, PBMCs, and mononuclear cells from MLN were stained with cell surface markers for 30 min at 4°C, followed by dead cell staining. CD4^+^ 7AAD^−^ cells were then sorted on BD FACSAria. Cells were then incubated with Human TruStain FcX (BioLegend) for 10 min at 4°C and labeled with TotalSeq-C Human Universal Cocktail, V1.0 (#399905; BioLegend), for 30 min at 4°C. Subsequently, cells were resuspended in PBS with 0.04% BSA at a density of 1,000 cells/μl. Libraries for sequencing were generated following the same protocol as described for scRNA-seq library preparation for CD103^+^ CD4^+^ T cell data.

### CITE-seq library preparation for TCR data

CD4^+^ T cells from colonic mucosa, MLN, and PBMCs were isolated as described above. CD4^+^ T cells were sorted using FACS and hashtagged as described in scRNA-seq library preparation section for CD103^+^ CD4^+^ T cell data. After hashtagging, cells were stained with TotalSeq-C Human Universal Cocktail, V1.0 (BioLegend), or TotalSeq-C0145 anti-human CD103 antibody (BioLegend). Subsequently, cells were resuspended in PBS with 0.04% BSA at a density of 1,000 cells/μl. Libraries for sequencing were generated following the same protocol as described for scRNA-seq library preparation section for CD103^+^ CD4^+^ T cell data.

### CITE-seq and scRNA-seq data processing

The 10x Genomics Cell Ranger (v7.2) pipeline was used to process the CITE-seq and scRNA-seq data. For scRNA-seq data, reads were aligned to the GRCh38 human reference genome and gene expression matrices were generated using “cellranger count.” For CITE-seq data, “cellranger multi” was utilized to obtain gene expression, ADT counts, and TCR sequences. The raw RNA count matrix and ADT data were further processed using the R package Seurat (v5.1.0). Filtering based on RNA-assay metrics (200 < nFeature_RNA < 7,000, percent.mt < 15) resulted in 34,715 cells for CITE-seq and 12,454 cells for CD103^+^ CD4^+^ T cell scRNA-seq. The gene expression count matrix was then normalized using the NormalizeData function. PCA was based on the top 2,000 highly variable features.

### CITE-seq data analysis

Anchor-based RPCA integration was used to integrate multiple sequencing runs for RNA data. Cell annotations were made based on protein and RNA expression data. UMAP was generated using the first 30 dimensions. We used Milo (v1.2.0) to test for the differential abundance of cells within defined neighborhoods, between two conditions (control and CD). For single-cell GSEA, gene sets were generated from the top 100 genes from the lentiviral RUNX2 and BHLHE40 overexpression data. Enrichment scores were evaluated using the escape (v1.99.1) R package. Reference mapping of scRNA-seq and scMultiome data was performed using the Seurat package (v5.1.0). Our dataset (query) was preprocessed and normalized as described above, and the published reference dataset from CD4^+^ T cells in the human colon (Yokoi et al., 2023) was also preprocessed and normalized as described in the article. To identify anchors between the reference and query datasets, we used the FindTransferAnchors function with default parameters (normalization.method = “SCT,” reference.reduction = “pca,” dims = 1:30). Cell-type labels and UMAP embeddings from the published reference were transferred to our dataset using the MapQuery function, based on the identified anchors.

### scMultiome data generation

CD4^+^ T cells were collected from fresh samples using the same method as for CITE-seq. After sorting CD4^+^ T cells, the cells were centrifuged at 500 *g* for 5 min at 4°C. The cell pellets were resuspended in 100 μl of chilled 0.1× lysis buffer (10 mM Tris-HCl, pH 7.4, 10 mM NaCl, 3 mM MgCl_2_, 1% BSA, 1 mM DTT, 1 U/μl RNase inhibitor, 0.01% Tween-20, 0.01% Nonidet P-40 substitute, and 0.001% digitonin) and incubated on ice for 7 min. The lysis reaction was stopped by adding 1 ml of chilled wash buffer (10 mM Tris-HCl, pH 7.4, 10 mM NaCl, 3 mM MgCl_2_, 1% BSA, 1 mM DTT, 1 U/μl RNase inhibitor, and 0.1% Tween-20). The cells were then collected by centrifugation at 500 *g* for 5 min at 4°C and washed twice with 1 ml of wash buffer. During the second wash, cells were filtered through a 40-µm FLOWMI cell strainer. Nuclei were resuspended at a concentration of 3,200 nuclei/μl in diluted nuclei buffer (1× nuclei resuspension buffer, 1 mM DTT, and 1 U/μl RNase inhibitor). Single-cell ATAC/RNA-seq libraries were constructed using the Chromium Next GEM Single Cell Multiome ATAC + Gene Expression Reagent Bundle (10x Genomics) according to the manufacturer’s instructions. The libraries were sequenced on a NovaSeq 6000 (Illumina) with a read length of 50 cycles (read 1), 10 cycles (i7 index), 24 cycles (i5 index), and 91 cycles (read 2).

### scMultiome data processing

The 10x Genomics Cell Ranger ARC (v2.0.0) pipeline was used to process the Multiome data. The raw files of RNA-seq and ATAC-seq libraries from the same sample were aligned to the UCSC Human Genome (hg38) and quantified using “cellranger-arc count.” Samples were aggregated using “cellranger-arc aggr” to normalize the sequencing depth. The raw RNA count matrix and ATAC fragment data were further processed using R packages Seurat (v5.1.0) and Signac (v1.13.0), respectively. Filtering based on RNA-assay metrics (500 < nCount_RNA < 30,000, nFeature_RNA < 7,500, percent.mt < 25) and ATAC-assay metrics (500 < nCount_ATAC < 100,000, nucleosome_signal < 2, TSS.enrichment > 1) resulted in 42,553 cells. The gene expression count matrix was then normalized using the NormalizeData function. PCA was based on the top 2,000 highly variable features. For the ATAC data, peak calling was performed using the MACS2 package with CallPeaks function in Signac. Peaks overlapping with genomic blacklist regions for the hg38 genome were removed. The peak count matrix was then normalized using latent semantic indexing (LSI), which includes term frequency–inverse document frequency and singular value decomposition. The first LSI component, highly correlated with sequencing depth, was removed from the downstream analysis.

### scMultiome data analysis

Anchor-based CCA integration was used to integrate multiple sequencing runs for RNA and ATAC data. Weighted nearest neighbor analysis from the Seurat package and Harmony was used to generate the UMAP (dimensions = 1:30 for RNA and dimensions = 2:30 for ATAC) and clusters (resolution = 0.8, algorithm = 3). The MapQuery function from the Seurat package was used for reference mapping, and the highest prediction score was chosen for annotation. Clusters with the same annotation were combined for further analysis. Differentially expressed genes and differentially accessible open chromatin regions in each cluster were identified using the FindMarkers function. TF enrichment analysis was performed using ChIP-Atlas. Motif analysis was performed using the FindMotifs function from Signac. For KD simulation, the CellOracle package was used.

### TCR repertoire analysis

Clonotypes were added to the integrated Seurat object using the scRepertoire (v2.0.4) R package. Circos visualization was performed using the circlize (v0.4.16) R package. The UpSet plot was generated using the UpSetR (v1.4.0) R package.

### Vector construction for lentiviral overexpression

Total RNA was extracted from human colon LPMC using FAST Gene RNA Premium Kit (Nippon Genetics), and cDNA was synthesized using ReverTra Ace qPCR RT Master Mix (TOYOBO). *RUNX2* and *BHLHE40* were amplified using the following primers: *RUNX2*, 5′-GGT​GTC​GTG​ACG​TAC​GGC​CAC​CAT​GCG​TAT​TCC​CGT​AGA​TCC​G-3′ and 5′-CTC​CAC​TGC​CGC​TAG​CAT​ATG​GTC​GCC​AAA​CAG​ATT​CAT​C-3′; *BHLHE40*, 5′-GGT​GTC​GTG​ACG​TAC​GGC​CAC​CAT​GGA​GCG​GAT​CCC​CAG​C-3′ and 5′-CTC​CAC​TGC​CGC​TAG​CGT​CTT​TGG​TTT​CTA​AGT​TTA​AAG​GGG​GGA​TT-3′; RUNX1, 5′-AAA​ACG​TAC​GGC​CAC​CAT​GGC​TTC​AGA​CAG​CAT​ATT​TGA​G-3′ and 5′-AAA​AGC​TAG​CGT​AGG​GCC​TCC​ACA​CGG-3′; and RUNX3, 5′-AAA​ACG​TAC​GGC​CAC​CAT​GGC​ATC​GAA​CAG​CAT​CTT​C-3′ and 5′-AAA​AGC​TAG​CGT​AGG​GCC​GCC​ACA​CG-3′. The amplicon was cloned into the pXR001 (#109049; Addgene) using restriction enzymes BsiWI and NheI and DNA Ligation Kit (Takara Bio).

### Vector construction for CRISPR KD and KO

The EF1α core promoter from lentiCRISPR v2-dCas9 (#112233; Addgene) was replaced with a MSCV promoter fragment (IDT), and the Cas9-P2A-Puromycin cassette was substituted with gene fragments (IDT) encoding dCas9-ZIM3-T2A-mScarlet using the Gibson assembly. Oligonucleotides for gRNA sequences were purchased from Thermo Fisher Scientific: control, 5′-CAC​CGT​GTC​TTT​AAA​CAC​GCC​ATC​G-3′ and 5′-AAA​CCG​ATG​GCG​TGT​TTA​AAG​ACA​C-3′; RUNX2 KD, 5′-CAC​CGG​GCG​GGT​AGG​GAG​ACC​CGG​G-3′ and 5′-AAA​CCC​CGG​GTC​TCC​CTA​CCC​GCC​C-3′; BHLHE40 KD, 5′-CAC​CGA​CGG​CGC​AGA​CAG​ACC​GCG​C-3′ and 5′-AAA​CGC​GCG​GTC​TGT​CTG​CGC​CGT​C-3′; RUNX1 KO, 5′-CAC​CGT​GCT​CCC​CAC​AAT​AGG​ACA​T-3′ and 5′-AAA​CAT​GTC​CTA​TTG​TGG​GGA​GCA​C-3′; RUNX3 KO, 5′-CAC​CGA​AGC​GGC​TCT​CCG​TGA​GGG​T-3′ and 5′-AAA​CAC​CCT​CAC​GGA​GAG​CCG​CTT​C-3′; TBX21 KD, 5′-CAC​CGG​GTC​CTC​GAC​GGC​TAC​GGG​A-3′ and 5′-AAA​CTC​CCG​TAG​CCG​TCG​AGG​ACC​C-3′; RBPJ KD, 5′-CAC​CGA​GAT​GGC​GCC​TGT​TGT​GAC​A-3′ and 5′-AAA​CTG​TCA​CAA​CAG​GCG​CCA​TCT​C-3′. The oligonucleotides were annealed by mixing 1 μl forward strand oligo, 1 μl reverse strand oligo, 7 μl nuclease-free water, 1 μl 10×TNE buffer (100 mM Tris-HCl, pH 8.0, 10 mM EDTA, and 500 mM NaCl), and heated to 95°C for 5 min and then allowed to cool to RT. DNA ligation was performed using DNA Ligation Kit (Takara Bio) according to the manufacturer’s protocol.

### Lentivirus production

Lenti-X293T cells were seeded at 7 × 10^6^ cells onto collagen-treated 10-cm dishes in viral harvest medium (Opti-MEM I Reduced Serum Medium [Gibco] supplemented with 5% FBS and 100 mM sodium pyruvate) on day 1. On day 2, cells were transfected with 3.8 µg VSVg, 5.85 µg psPAX2, 7.6 µg of the transgene lentivirus vector using Opti-MEM I Reduced Serum Medium, and Lipofectamine 3000 (Thermo Fisher Scientific). After 6 h, the culture medium was replaced with viral harvest medium containing viral boost reagent (ALSTEM). On days 3 and 4, the viral supernatant was collected and stored at 4°C. For concentration of the virus, the viral supernatant was centrifuged at 500 *g* for 10 min at 4°C and the supernatant was mixed with the Lentiviral Precipitation Solution (ALSTEM) and incubated overnight at 4°C. The next day, the mixed supernatant was centrifuged at 1,500 *g* for 40 min at 4°C and the supernatant was removed. The virus-containing pellet was resuspended in cold PBS and stored at −80°C until use.

### Lentiviral overexpression

CD4^+^ T cells from the peripheral blood were stained with antibodies against CD3-APC/Cy7 (#300426; BioLegend), CD4-PE/Cy7 (#557852; BD Biosciences), and CD45RA-APC (#304112; BioLegend) for 30 min at 4°C and washed two times with FACS buffer. For dead cell staining, cells were resuspended with FACS buffer containing 7AAD (#420404; BioLegend) and acquired on BD FACSMelody (BD Biosciences). CD3^+^ CD4^+^ CD45RA^+^ 7AAD^−^ T cells were used for lentiviral overexpression. On day 0, 96-well plates were incubated with anti-hCD3 (#300438; BioLegend) at 10 µg/ml and anti-hCD28 antibodies (#302933; BioLegend) at 5 µg/ml for 3 h. After washing twice with RPMI-1640 supplemented with 2% FBS, 1.0 × 10^5^ naïve CD4^+^ T cells were seeded onto the anti-CD3/CD28-coated plate. T cells were cultured in RPMI-1640 medium containing D-glucose and glutamine supplemented with 10% FBS, 100 U/ml penicillin, 100 µg/ml streptomycin sulfate (Thermo Fisher Scientific), 10 mmol/l HEPES (NACALAI TESQUE), 100 mM sodium pyruvate (NACALAI TESQUE), and 100 U/ml hIL-2 (KYOWA Pharmaceutical Industry Co.). On day 1, the control-GFP, control-BFP, RUNX2-GFP, BHLHE40-BFP, RUNX1-GFP, and RUNX3-GFP lentiviruses were added to the culture medium and incubated at 37°C in 5% CO_2_ for 10 min. The culture plates were then centrifuged at 1,200 *g* for 90 min at 32°C and returned to the incubator. On day 3, the cells were passaged to a 24-well plate. On day 5, the T cells were collected for analysis.

### Lentiviral KD

Isolated LPMC were stained with antibodies against CD3-APC/Cy7 (#300426; BioLegend) and CD4-PE/Cy7 (#557852; BD Biosciences) for 30 min at 4°C and washed two times with FACS buffer. For dead cell staining, cells were resuspended with FACS buffer containing 7AAD (#420404; BioLegend) and acquired on BD FACSMelody (BD Biosciences). CD3^+^ CD4^+^ 7AAD^−^ T cells were used for lentiviral KD and KO experiments. Cells were seeded to a CD3/CD28-coated 96-well plate and infected with RUNX2 KD, BHLHE40 KD, TBX21 KD, RBPJ KD, RUNX1 KO, and RUNX3 KO lentiviruses as described in the overexpression experiment. On day 3, the culture medium was replaced with fresh medium. On day 5, T cells were collected and mScarlet3-positive cells were collected by FACS.

### Western blotting

CD4^+^ T cells and Jurkat cells were lysed in RIPA buffer (Thermo Fisher Scientific) supplemented with protease inhibitor cocktail (#11836153001; Roche) on ice for 15 min, followed by centrifugation at 14,000 *g* for 15 min at 4°C. Supernatants were collected and mixed with sample buffer solution with reducing reagent (NACALAI TESQUE), and protein samples were separated by SDS–PAGE and transferred onto PVDF membranes (Millipore). Membranes were blocked in 5% nonfat dry milk in Tris-buffered saline with 0.1% Tween-20 (TBS-T) for 1 h at RT and incubated with primary antibodies: anti-AML1 (RUNX1) antibody (#4334; Cell Signaling Technology), anti-RUNX3 antibody (#564813; BD Pharmingen), and anti-GAPDH antibody (#sc-32233; Santa Cruz Biotechnology), at a 1:1,000 dilution overnight at 4°C. After washing three times with TBS-T, membranes were incubated with HRP-conjugated secondary antibodies at a 1:3,000 dilution for 1 h at RT. Signal was developed using a ECL substrate (#11644-40; NACALAI TESQUE) and visualized using a chemiluminescence detection system (ImageQuant LAS500; Cytiva).

### Quantitative PCR

Total RNA was isolated using Fast Gene RNA Premium Kit (NIPPON Genetics); the RNA was reverse-transcribed with ReverTra Ace qPCR RT Master Mix with gDNA Remover (TOYOBO). For KD experiment with lamina propria CD4^+^ T cells, 500 cells were collected, and RNA isolation and cDNA synthesis were performed using QuantAccuracy, RT-RamDA cDNA Synthesis Kit (TOYOBO). Real-time qPCR was performed using StepOnePlus Real-Time PCR System (Applied Biosystems) using Power SYBR Green PCR Master Mix (Applied Biosystems). All values were normalized to the expression of *GAPDH*, and the relative fold change in expression compared with *GAPDH* was evaluated. The amplification conditions were 50°C (2 min), 95°C (10 min), and 40 cycles of 95°C (15 s) and 64°C (60 s). The following primer sets were used: *GAPDH*: 5′-GTC​GGA​GTC​AAC​GGA​TT-3′ and 5′-AAG​CTT​CCC​GTT​CTC​AG-3′; *RUNX2* variant1: 5ʹ-ATG​CGT​ATT​CCC​GTA​GAT​CC-3ʹ and 5ʹ-GGG​CTC​ACG​TCG​CTC​ATT​T-3ʹ; *RUNX2* variant2: 5ʹ-AGG​AGG​GAC​TAT​GGC​ATC​AAA​C-3ʹ and 5ʹ-GGG​CTC​ACG​TCG​CTC​ATT​T-3ʹ; *BHLHE40*: 5ʹ-TAA​AGC​GGA​GCG​AGG​ACA​GCA​A-3ʹ and 5ʹ-GAT​GTT​CGG​GTA​GGA​GAT​CCT​TC-3ʹ; *IFNG*: 5ʹ-TCC​CAT​GGG​TTG​TGT​GTT​TA-3ʹ and 5ʹ-AAG​CAC​CAG​GCA​TGA​AAT​CT-3ʹ; *GZMB*: 5ʹ-CGA​CAG​TAC​CAT​TGA​GTT​GTG​CG-3ʹ and 5ʹ-TTC​GTC​CAT​AGG​AGA​CAA​TGC​CC-3ʹ; *PRF1*: 5ʹ-ACT​CAC​AGG​CAG​CCA​ACT​TTG​C-3ʹ and 5ʹ-CTC​TTG​AAG​TCA​GGG​TGC​AGC​G-3ʹ; *S1PR1*: 5ʹ-CCT​GTG​ACA​TCC​TCT​TCA​GAG​C-3ʹ and 5ʹ-CAC​TTG​CAG​CAG​GAC​ATG​ATC​C-3ʹ; *RBPJ*: 5′-TCA​TGC​CAG​TTC​ACA​GCA​GTG​G-3′ and 5′-TGG​ATG​TAG​CCA​TCT​CGG​ACT​G-3′.

### Bulk RNA-seq

For *RUNX2* and *BHLHE40* overexpression experiments, CD4^+^ T cells transduced with lentiviral vectors were stimulated with PMA (10 ng/ml) and ionomycin (250 ng/ml) for 2 h, and washed with FACS buffer, followed by staining with antibodies against CD3-APC/Cy7 (#300426; BioLegend) and CD4-PE/Cy7 (#557852; BD Pharmingen). After dead cell staining, GFP^+^ BFP^+^ 7AAD^−^ CD4^+^ T cells were sorted using FACSMelody. For comparison between HLA-DR^+^ CD4^+^ T_RM_ and HLA-DR^−^ CD4^+^ T_RM_, colonic LPMC were incubated with PMA (10 ng/ml) and ionomycin (250 ng/ml) for 2 h at 37°C, and washed in FACS buffer twice, followed by staining with antibodies against CD3-APC/Cy7 (#300426; BioLegend), CD4-PE/Cy7 (#557852; BD Pharmingen), and HLA-DR-APC (#559666; BD Pharmingen) for 30 min at 4°C. Cells were then washed with FACS buffer; HLA-DR^+^ and HLA-DR^−^ T_RM_ were sorted using FACSMelody. Isolated cells were then lysed with TRIzol Reagent (Thermo Fisher Scientific), and total RNA was purified using miRNeasy Micro Kit (QIAGEN). Full-length cDNA and Illumina libraries were generated using SMART-Seq mRNA HT LP. Sequencing was performed on a NovaSeq 6000 platform (Illumina) in 101-base single-read mode. Generated reads were mapped to the human (hg19) reference genome using TopHat v2.1.1 with Bowtie2 v2.2.8 and SAMtools v0.1.18. Read count data were analyzed using iDEP.96 (Ge et al., 2018; Ge, 2021). Hierarchical clustering and PCA were performed. Differentially expressed genes (false discovery rate <0.1) were then selected using DESeq2 (Love et al., 2014). GSEA was performed with GSEA software v4.3.2 using the c5.cc gene set collection (MSigDB v6.2).

### CUT&RUN assay

Chromatin profiles in human PBMC-derived T cells overexpressing RUNX2 or BHLHE40 through lentiviral infection were assayed using a ChIC/CUT&RUN assay kit (#53180; Active Motif) following the manufacturer’s protocol. Briefly, T cells were stimulated with PMA (10 ng/ml) and ionomycin (250 ng/ml) and incubated at 37°C for 2 h. After stimulation, 4 × 10^5^ cells per sample were harvested and washed with Complete Dig-Wash Buffer. Cells were then bound to concanavalin A beads, incubated for 10 min at RT, and nutated at 30 rpm, 4°C overnight with 2 µg of the following antibodies: normal rabbit IgG (#2729; Cell Signaling Technology), anti-RUNX2 (D1L7F) antibody (#12556; Cell Signaling Technology), anti-SHARP2/DEC1 antibody (#70723; Abcam), and anti-acetyl-histone H3 (Lys27) antibody (#4353; Cell Signaling Technology) in 100 μl antibody buffer (final concentration of 0.5% digitonin) per sample. The next day, beads were washed with cell permeabilization buffer and were bound to ChIC/CUT&RUN pAG-MNase, and incubated for 10 min at RT. Beads were collected and washed again with cell permeabilization buffer, and then, 100 mM of calcium chloride was added on ice to activate the enzymatic reaction. Chromatin digestion was performed at 4°C for 2 h of nutation at 30 rpm. Digestion was then stopped by Stop solution, and DNA fragments were released in solution after incubation at 37°C for 10 min. DNA fragments were isolated and purified using DNA purification columns and proceeded to NGS library preparation using NEBNext Ultra II DNA Library Preparation for Illumina (New England Labs) to prepare sequencing libraries according to the manufacturer’s protocol, and then, sequencing on Illumina NovaSeq 6000 was performed. CUT&RUN sequencing was performed and analyzed with paired RNA-seq data. Fastq files were trimmed using trimmomatic v0.39 (Bolger et al., 2014) and aligned to the hg38 genome via Bowtie2 v2.5.4 (Langmead and Salzberg, 2012). SAM files were converted to BAM files using SAMtools v1.6 (Danecek et al., 2021), and PCR duplicates were removed using picard v2.20.4 (https://broadinstitute.github.io/picard/). BigWig files were generated using the bedtools v2.31.1 bamCoverage function (Quinlan and Hall, 2010). MACS v2.2.9.1 (Zhang et al., 2008) was used to call peaks in each sample relative to IgG control background (parameters -f BAMPE, -g hs, -q 0.05 and --keep-dup -all). The CUT&RUN-seq peaks were analyzed in IGV.

### Study approval

Participants were recruited as part of routine clinical practice, and no selection of eligible patients was performed. All of the samples used in this study were collected with informed consent from patients who underwent surgery for either colon cancer or CD at the Department of Gastroenterological Surgery, Graduate School of Medicine, The University of Osaka, and at the Division of Inflammatory Bowel Disease Surgery, Department of Gastroenterological Surgery, Hyogo Medical University. This study was approved by the Ethical Committees of The University of Osaka School of Medicine (549, 15,435) and Hyogo Medical University (0407), and written informed consent for specimen use was obtained from all patients.

### Statistical analysis

Statistical analyses were performed using GraphPad Prism (GraphPad Software) and/or R unless otherwise noted. The statistical test used in the study and P values are specified in each figure legend. Outliers were identified using the interquartile range (IQR) method. A data point was considered an outlier if it was smaller than Q1 (the first quartile) − 1.5 × IQR or greater than Q3 (third quartile) + 1.5 × IQR. This method was applied independently to each group or condition to avoid bias introduced by pooling heterogeneous distributions.

### Online supplemental material

Fig. S1 shows the detailed information on the CITE-seq data used in Fig. 1. Fig. S2, A and B, shows the TCR clonality of each sample shown in Fig. 3. Fig. S2, C–E, presents the TCR information corresponding to Fig. 2. Fig. S3, A–E, shows detailed information on the scMultiome data used in Fig. 5. Fig. S3, F–I, provides additional data related to Fig. 7. Fig. S4 shows gating strategy of cell sorting, difference in *RUNX2* expression by variants, and the effect of RUNX2 and BHLHE40. Fig. S5, A–C, shows additional information on RUNX2 and BHLHE40 KD experiments from Fig. 8. Fig. S5, D and F, provides data on RUNX1 and RUNX3 KO experiments. Table S1 contains the clinical and demographic information for the human samples used in this study. Table S2 lists differentially expressed genes in each cluster identified from the CITE-seq data. SourceData FS5 provides the raw data for Fig. S5 D.

## Supplementary Material

Table S1 contains the clinical and demographic information for the human samples used in this study.

Table S2 lists differentially expressed genes in each cluster identified from the CITE-seq data.

SourceData FS5 is the source file for Fig. S5.

## Data Availability

All data produced by this study are available under the following accession numbers: GSE281504 for CITE-seq, GSE281509 for CD4^+^ CD103^+^ T cell scRNA-seq, GSE280714 for scMultiome, GSE301689 for scRNA-seq and TCR-seq of CD4^+^ T cells from colonic mucosa, MLN, and blood, GSE275362, GSE300848, and GSE300850 for bulk RNA-seq, and GSE303141 for CUT & RUN analysis. All scripts and data used for scRNA-seq analysis in this study are available through the GitHub repository (https://github.com/m-arase/TRMproject) and archived on Zenodo (https://doi.org/10.5281/zenodo.16598968).
