# Supplementary figures and images for: Multi-omics uncovers transcriptional programs of gut-resident memory CD4+ T cells in Crohn’s disease
Source: J Exp Med. 2025 Sep 4;222(11):e20242106. doi: 10.1084/jem.20242106 (PMC12410336; doi:10.1084/jem.20242106)

Fig. S5.-D Source Data

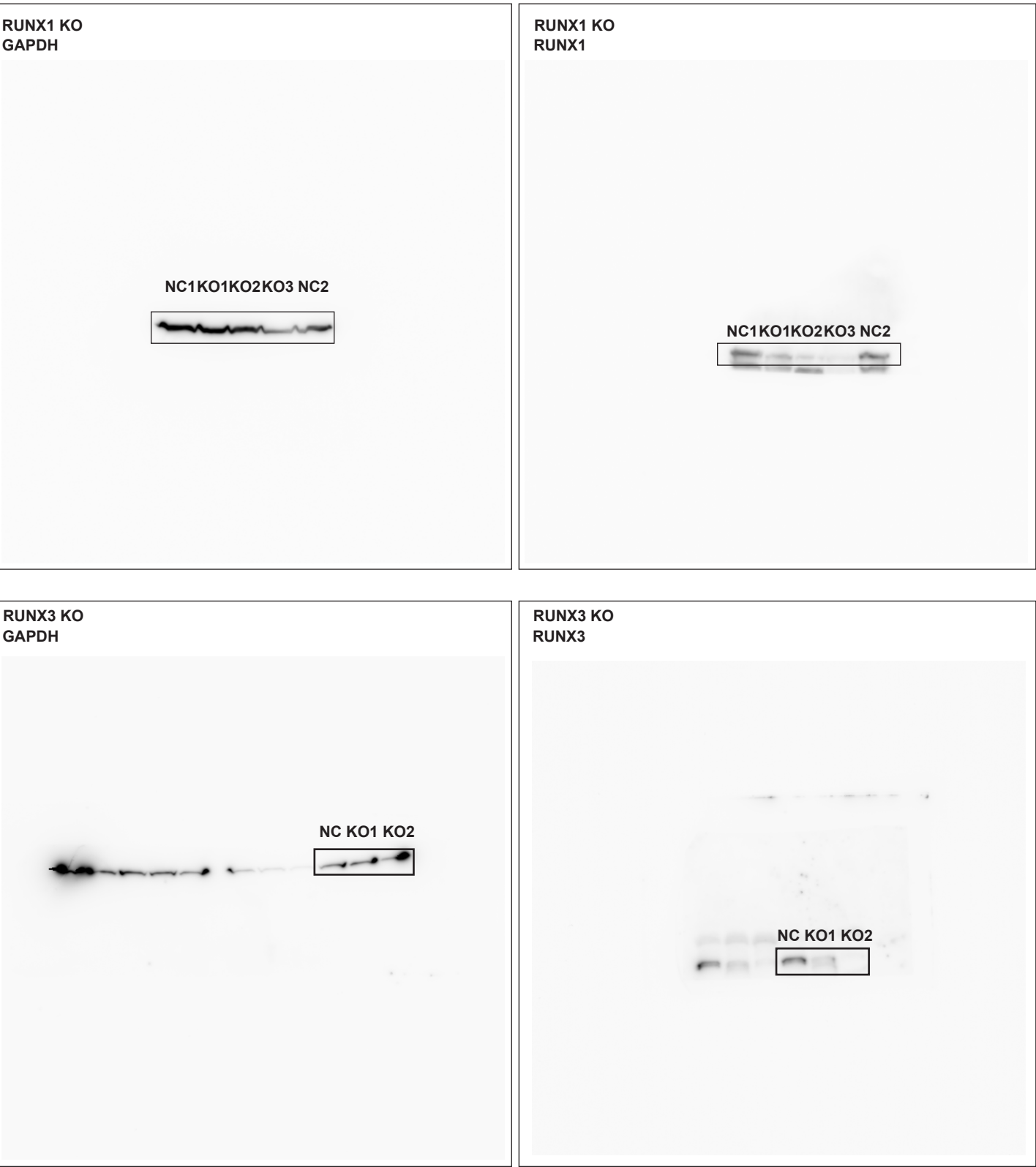

Supplement: SourceData FS5 — is the source file for Fig. S5. [file jem_20242106_sourcedatafs5.pdf]
